# In Silico Perturbation Identifies Transcription Factors as Protective Targets in HSPCs After Irradiation

**DOI:** 10.3390/ijms27083522

**Published:** 2026-04-15

**Authors:** Zongjian Tao, Qi Zhang, Yingying Chen, Shaoting Lv, Qilin Huang, Hongyue Tian, Qixiang Liu, Caihui Li, Yuyuan Wang, Hao Lu, Cheng Quan, Hongxia Chen, Yiming Lu, Gangqiao Zhou

**Affiliations:** 1 Academy of Military Medical Sciences, Beijing 100850, China; zongjiant@126.com (Z.T.); zqzq0316@163.com (Q.Z.); chenyingying0525@163.com (Y.C.); lvsttt0429@163.com (S.L.); huangqilin98@163.com (Q.H.); tianhyws@126.com (H.T.); lqixiang123@163.com (Q.L.); 18730833100@163.com (C.L.); luh678@163.com (H.L.); quanc1989@163.com (C.Q.); chenhongxia0626@163.com (H.C.); 2 College of Life Sciences, Hebei University, Baoding 071002, China; 3 School of Public Health, Nanjing Medical University, Nanjing 210023, China; 4 College of Traditional Chinese Medicine, Tianjin University of Traditional Chinese Medicine, Tianjin 301617, China; 5 College of Life Sciences, Zhejiang University, Hangzhou 310058, China; 3220105874@zju.edu.cn

**Keywords:** radiation, hematopoietic stem and progenitor cells, scRNA-seq, transcriptional regulation, in silico perturbation, *Tcf7l2*, *Hsf1*

## Abstract

Hematopoietic stem and progenitor cells (HSPCs) in the bone marrow are highly vulnerable to radiation-induced damage. Systematic delineation of lineage-specific transcription factor (TF) programs, together with in silico perturbation analyses, provides a valuable approach for identifying regulators capable of accelerating hematopoietic reconstruction after irradiation. Here, using single-cell RNA sequencing (scRNA-seq), we characterized the dynamics of HSPCs at both cellular abundance and transcriptional regulation levels following irradiation and used in silico TF perturbation to predict their effects on lineage commitment. We found that granulocyte–macrophage progenitor (GMP) differentiation is consistently prioritized after irradiation, accompanied by enhanced activity of proliferation-associated drivers. Network-based TF profiling identified *Tcf7l2* as a previously unrecognized regulator of early lymphoid differentiation. In silico perturbation further functionally predicted TFs driving differentiation in HSPCs after irradiation, and *Hsf1*, a factor with pharmacological activation potential, was selected for validation via in vivo celastrol treatment and in vitro knockdown. Collectively, our findings uncover the transcriptional programs governing HSPC lineage biases after radiation exposure and highlight the utility of in silico TF perturbation as a strategy for guiding the therapeutic interventions for radiation-induced hematopoietic injury.

## 1. Introduction

Radiotherapy or accidental radiation exposure can cause profound damage to the bone marrow (BM) and its hematopoietic stem and progenitor cells (HSPCs) [1,2], leading to hematopoietic acute radiation syndrome (H-ARS). This condition is characterized by severe myelosuppression and a markedly increased risk of infection, bleeding, and mortality [3]. Timely therapeutic intervention is therefore essential for mitigating life-threatening complications and promoting hematopoietic recovery.

Current therapeutic strategies for radiation-induced hematopoietic injury primarily rely on cytokine-based interventions that modulate HSPC differentiation [4]. Granulocyte colony-stimulating factor (G-CSF) is widely used to accelerate neutrophil regeneration [4], while thrombopoietin (TPO) facilitates megakaryocyte expansion and platelet recovery [5]. In addition, emerging stem cell-based therapies, such as mesenchymal stromal cell (MSC) transplantation, have shown promise in improving survival and supporting BM regeneration following irradiation [6,7]. However, these treatments act mainly through extrinsic signaling cues that only indirectly modulate the transcriptional programs governing lineage commitment, highlighting the need for developing strategies that directly target regulatory factors capable of reshaping hematopoietic recovery at its source.

Transcription factors (TFs) orchestrate hematopoietic cell fate decisions through their control of gene regulatory networks (GRNs) that integrate intrinsic and extrinsic signals. Genetic ablation studies have identified a series of master regulators that orchestrate the balance between HSC self-renewal and lineage-specific differentiation, including RUNX1, TAL1 (SCL), and MEIS1 for HSC self-renewal [8,9,10]; GATA1 for erythropoiesis and megakaryopoiesis [11]; SPI1 (PU.1) for myelopoiesis and early lymphoid priming [12,13]; CEBPA and GFI1 for granulocytic differentiation [14,15]; and IKZF1, TCF3, and LEF1 for lymphoid lineage commitment [16,17]. Recent advances in single-cell RNA sequencing (scRNA-seq) and computational biology have enabled a more systematic understanding of hematopoietic regulatory mechanisms [18]. For example, Buenrostro and colleagues leveraged integrated single-cell omics to delineate dynamic transcription factor activities and map enhancer regulatory networks across hematopoietic lineages [19]. Similarly, Gao et al. reconstructed transcriptional regulatory networks underlying HSC ontogeny and identified previously unrecognized regulators, such as SP3 and MAZ, that are essential for HSC specification [20]. Our recent work utilizes scRNA-seq to picture a dynamic single-cell transcriptomic landscape of HSPCs during IR-induced regeneration process and demonstrated nuclear factor erythroid 2-related factor 2 (NRF2) as a critical downstream functional gene for BMP4-BMPR2 signaling on HSCs to resist IR-induced damage [21]. Nonetheless, a systemic assessment of lineage-specific TFs throughout hematopoietic injury and recovery is still lacking.

Targeting TFs represents a promising strategy for mitigating radiation-induced hematopoietic injury. For example, Kim and colleagues demonstrated that the activation of NRF2 signaling enhances HSPC function and mitigates IR-induced myelosuppression and mortality [22]. Poulos and colleagues reported that the NF-κB is a critical TF-regulating HSC function both at steady state and after myelosuppressive irradiation and that the inhibition of NF-κB promotes improved HSC function and pan-hematopoietic recovery [23]. Together, these findings support the notion that targeting key TFs involved in hematopoiesis may offer an effective strategy to accelerate hematopoietic regeneration following radiation-induced injury.

To determine the consequences of TF-targeting interventions, traditional approaches rely on genetic perturbation experiments, such as knock-out or knock-in models [24,25]. While informative, these methods are time-consuming and limited in scalability. Recently, perturbation modeling has emerged as a computational framework capable of simulating cellular responses to genetic or pharmacological interventions using single-cell omics data [26,27]. Among these approaches, biologically informed methods such as CellOracle [28] and SCENIC+ [29] perform in silico perturbations on gene regulatory networks to model the effects of transcriptional regulation, thereby enabling large-scale screening of potential intervention targets for hematopoietic disease and injury.

Here, we delineated the differentiation dynamics of HSPCs following irradiation using scRNA-seq data and established an integrative workflow that combines transcriptional activity analysis with in silico perturbation modeling to identify lineage-driving TFs. Through this framework, we uncovered *Tcf7l2* as a previously unrecognized regulator of lymphoid differentiation. Among the TFs predicted by in silico perturbation, *Hsf1*, which has pharmacological activation potential, was selected for validation via in vivo celastrol treatment and in vitro knockdown.

## 2. Results

### 2.1. Temporal Lineage Bias Profiling Reveals Prioritized GMP Expansion During Post-Irradiation Hematopoietic Reconstruction

**Figure 1:**
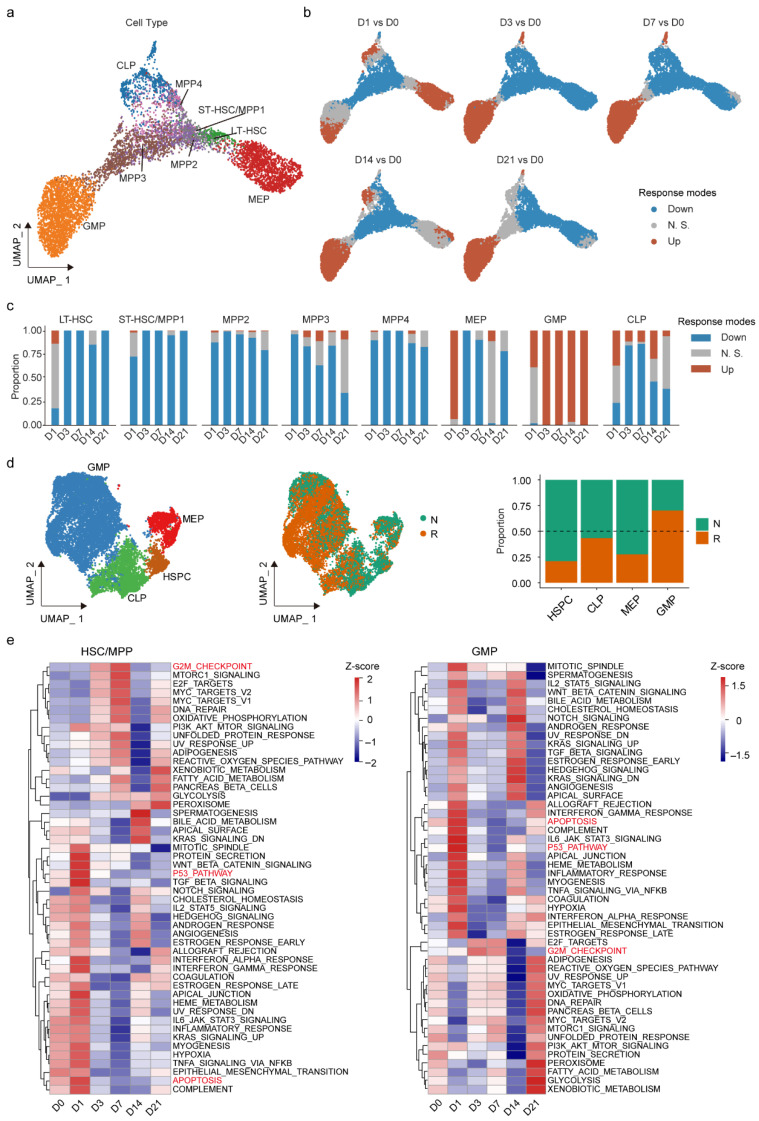
Dynamic responses of HSPCs following irradiation. (**a**) UMAP visualization of bone marrow HSPC subpopulations based on our previous HSPC subclustering results [21]. (**b**) MELD plots illustrating response modes of abundance (down, N.S. and up) at post-irradiation time points (D1, D3, D7, D14, and D21) relative to baseline (D0). (**c**) Stacked bar plot summarizing population-specific response modes in HSPCs after irradiation. (**d**) UMAP embedding of HSPCs from an independent scRNA-seq dataset (**left**), UMAP visualization of non-irradiated (N) and irradiated (R) samples at D3 (**middle**) and the corresponding stacked bar plot (**right**). (**e**) GSVA showing the enrichment of Hallmark pathways in HSC/MPP and GMP populations following irradiation. Cell cycle- and apoptosis-related pathways were highlighted in red. Enrichment scores were normalized by z-score.

**Figure A1:**
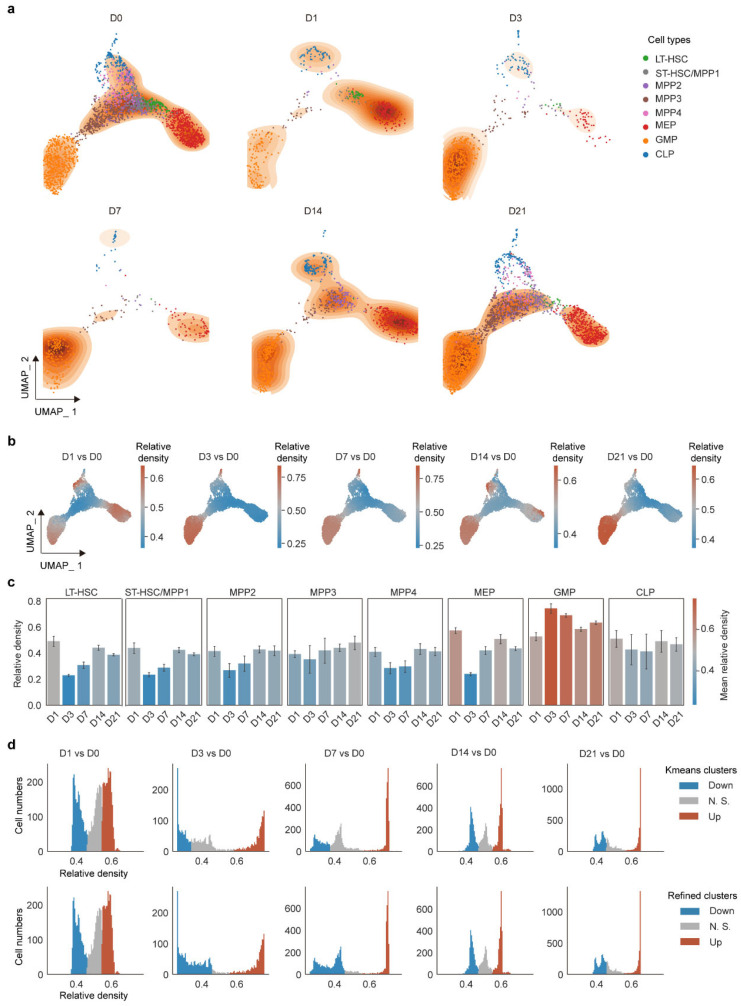
Dynamic abundance and classification of responses after irradiation. (**a**) Contour plots showing the abundance of HSPC subpopulations at pre-irradiation (D0) and post-irradiation time points (D1, D3, D7, D14, and D21). (**b**) UMAP visualization of MELD-inferred relative density at each post-radiation time point compared with D0. (**c**) Bar plots summarizing the relative density for each HSPC subpopulation across time. (**d**) Classification of response modes (down, N.S. and up) was first determined through unsupervised K-means clustering based on relative density (**top**), and it was subsequently refined using empirical thresholds (**bottom**).

To systemically characterize the differentiation biases in HSPCs after irradiation, we utilized the transcriptomic data of HSPCs in our scRNA-seq dataset of mouse BM [21], which includes single-cell transcriptomic data of eight HSPC subpopulations: LT-HSC, ST-HSC/MPP1, MPP2, MPP3, MPP4, MEP, GMP, and CLP (Figure 1a). The dataset comprises samples collected before irradiation (D0) and at five post-irradiation time points (D1, D3, D7, D14, and D21), revealing a complex temporal remodeling of population abundance (Figure A1a).

To quantify the dynamic responses of HSPC subpopulations post-irradiation, we applied MELD [30], a manifold-based likelihood estimation framework, to calculate the changes in abundance of each subpopulation compared to homeostatic status (D0) (Figure A1b,c). Unsupervised clustering, assisted by empirical thresholds, was then used to classify the populations into three response modes related to D0: down, no significance (N.S.), and up (Figure 1b and Figure A1d). The proportions of cells corresponding to the three modes exhibited distinct temporal patterns across different subpopulations of HSPCs (Figure 1c). All five HSC/MPP subpopulations (LT-HSC, ST-HSC/MPP1, MPP2, MPP3, and MPP4) exhibited sustained depletion 21 days after irradiation, consistent with the well-documented exhaustion of HSCs under stressful conditions [2], though LT-HSCs exhibited transient radioresistance on D1, they eventually succumbed to exhaustion (Figure 1c). On the other hand, the dynamics of MPPs have been less frequently described. Among committed progenitors, the MEP, GMP, and CLP populations exhibited a synchronized expansion at D1 (Figure 1c), reflecting a unified acute response to irradiation. However, from D3 onward, their responses diverged markedly. MEP and CLP underwent a depletion and recovery pattern (Figure 1c), with CLP additionally showing a transient shift toward more mature status during the depletion stage (Figure 1b). In contrast, GMPs displayed a persistent and progressive increase in density (Figure 1c).

**Figure A2:**
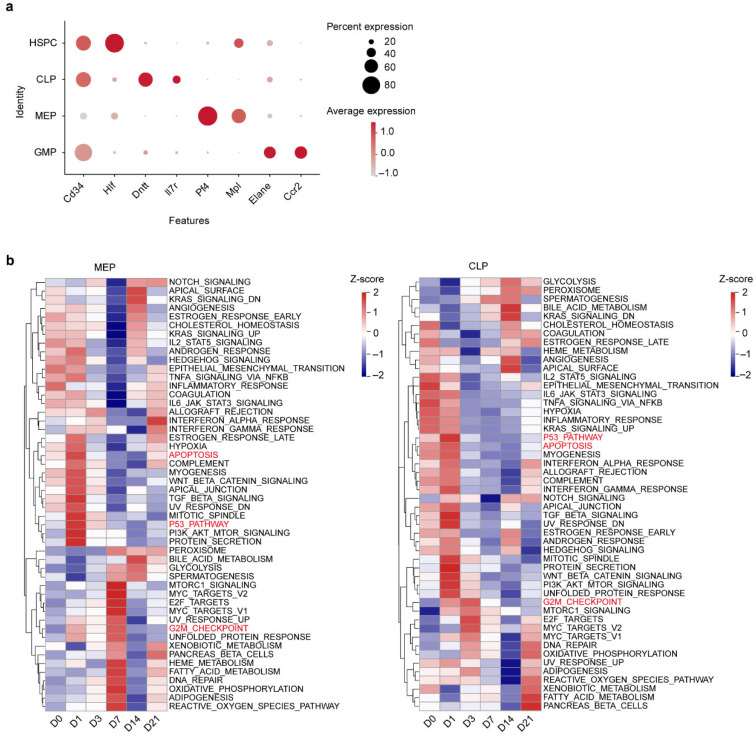
Additional analysis for dynamic responses after irradiation. (**a**) Dot plot showing cell type annotations in independent scRNA-seq dataset. (**b**) GSVA showing enrichment of Hallmark pathways in MEP and CLP populations following irradiation. Cell cycle- and apoptosis-related pathways were highlighted in red. Enrichment scores were normalized by z-score.

To validate this observation, we performed an additional scRNA-seq on BM hematopoietic cells at D0 and D3 after irradiation. After cell clustering and cell type annotation, we found the pronounced exhaustion of HSPCs and MEP, accompanied by an accumulation of GMP at D3, compared with non-irradiated controls (Figure 1d and Figure A2a, Table S1). To further investigate the alterations of hematopoietic progenitors at the molecular level, we calculated the activity scores of 50 hallmark pathways in MEP, GMP, and CLP at different time points along hematopoietic injury and reconstruction (Figure 1e and Figure A2b). Consistent with the abundance changes, the activities of p53 and apoptosis pathways peaked at D1 post-irradiation in all HSPC populations (Figure 1e), indicating an urgent stress immediately after irradiation. Notably, the G2M checkpoint pathway is immediately activated (D1) after irradiation in MEP, GMP, and CLP, while its activation in HSC/MPPs started from D3 post-irradiation (Figure 1e and Figure A2b), suggesting a more rapid response to irradiation for progenitor cells than HSC/MPPs. Together, these findings suggest distinct recovery priorities among MEP, GMP, and CLP after the rapid expansion at D1 post-irradiation and a preferential expansion of GMP from D3 post-irradiation.

### 2.2. SCENIC-Based TF Profiling Reveals Distinct Regulatory Modules and Identifies Tcf7l2 as a Lymphoid Regulator

**Figure 2:**
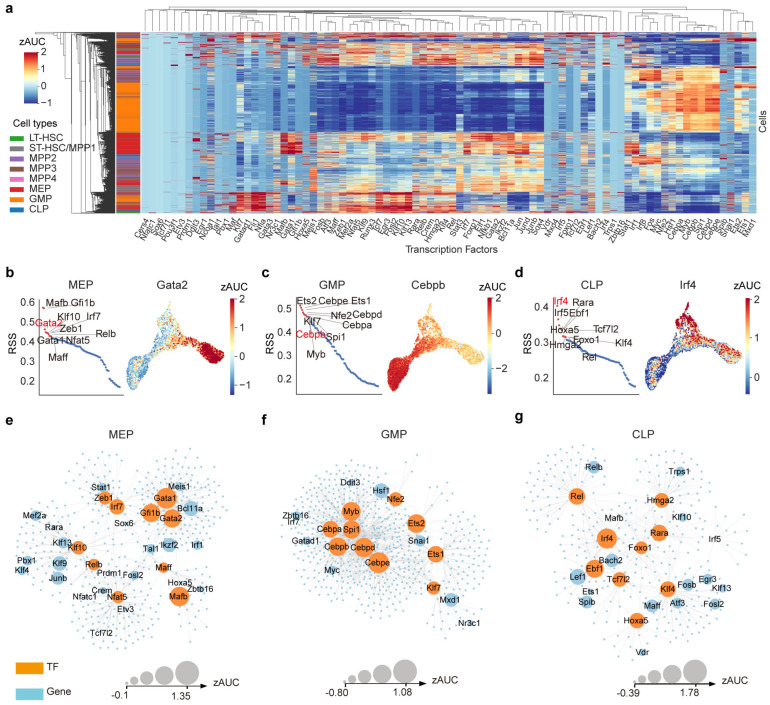
SCENIC analysis of transcriptional regulatory programs and identification of lymphoid function of *Tcf7l2*. (**a**) Hierarchical clustering and heatmap showing the distinct activity patterns across HSPC subpopulations. TF activity was calculated based on AUC (area under the curve) and normalized by z-score. (**b**–**d**) Scatter plots showing the top 10 TF markers ranked by RSS for MEP, GMP, and CLP. RSS, Regulon specificity score. The activity distribution of a representative TF (highlighted in red) was visualized on UMAP. (**e**–**g**) Specific regulatory networks for MEP, GMP, and CLP. Orange nodes represent TF markers, and blue nodes represent their target genes; node size corresponds to TF activity (z-scored AUC).

**Figure A3:**
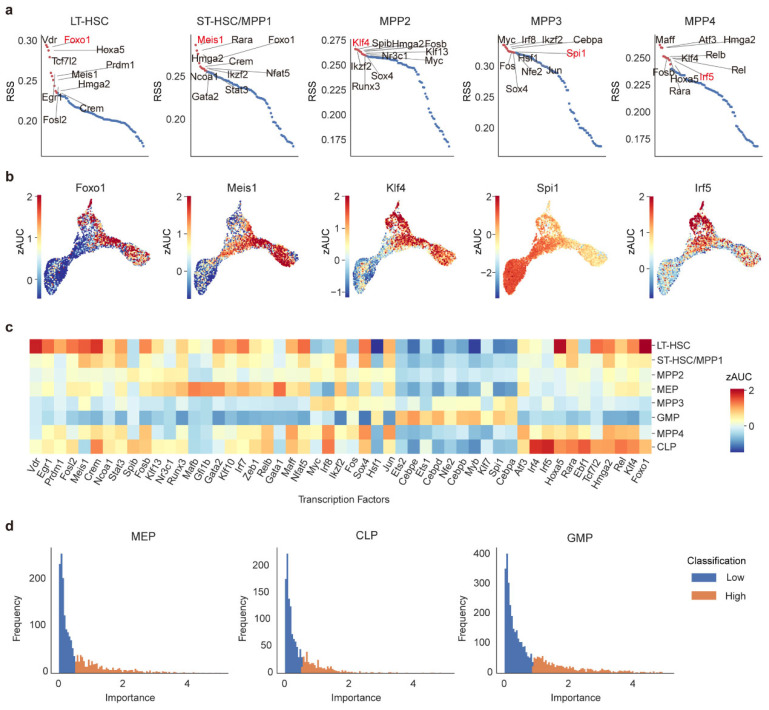
Additions for transcriptional regulatory analysis by SCENIC. (**a**,**b**) Scatter plots of the top 10 TF markers ranked by RSS for LT-HSC, ST-HSC/MPP1, MPP2, MPP3, and MPP4. The activity distribution of a representative TF (highlighted in red) was visualized on UMAP. (**c**) Heatmap showing the activity patterns of specific TFs across HSPC subpopulations. (**d**) Filtering of high-confidence interactions for constructing specific regulatory networks. TF–target interactions were classified by unsupervised clustering based on importance scores to keep high-confidence edges.

Having characterized the dynamic responses of distinct HSPC subpopulations post-irradiation, we next aimed to dissect the cell type-specific transcriptional regulatory programs. To this end, we performed a TF profiling analysis using SCENIC [31,32], a computational framework for regulatory network inference and regulon activity analysis (Figure 2a). To identify regulators enriched in specific cell types, we ranked TFs according to their regulon specificity score (RSS) and selected the top 10 TFs for each cell type as markers (Figure 2b–d and Figure A3a). The representative regulators exhibited cell type–restricted activity patterns on the UMAP projection (Figure 2b–d and Figure A3b). Notably, TF signatures were particularly specific in HSC, GMP, MEP, and CLP, whereas MPPs displayed activity profiles resembling those of their downstream lineages (Figure A3c).

To illustrate cell type-specific regulatory interactions, we constructed specific transcriptional regulatory networks for MEP, GMP, and CLP using markers and key regulatory relationships identified by Gaussian Mixture Models (Figure 2e–g and Figure A3d, Tables S2–S5). In the MEP network, canonical erythroid regulators such as *Gata1*, *Gata2* [33], and *Gfi1b* [34] formed a coherent regulatory module consistent with their roles in driving erythropoiesis (Figure 2e). Within the GMP network, core myeloid TFs, including the CEBP family [35,36] and *Spi1* [12], together with the proliferation regulators *Myb* [37] and *Ets1/2* [38,39], were organized into a densely interconnected regulatory hub that controls a shared set of targets (Figure 2f). The CLP network also incorporated lymphoid-associated TFs, including *Irf4* [40], *Irf5* [41], *Klf4* [42], and *Ebf1* [43] (Figure 2g). Notably, *Tcf7l2* appeared as a highly connected node, sharing strong regulatory links with *Irf4*, *Ebf1* and *Klf4* (Figure 2g), suggesting a previously unrecognized function in lymphoid development. As a member of the TCF family, *Tcf7l2* can form a bipartite transcription factor and influence multiple biological pathways, including the Wnt signaling pathway [44]. Here, our data suggest that *Tcf7l2* may also play a role in lymphoid differentiation.

**Figure 3:**
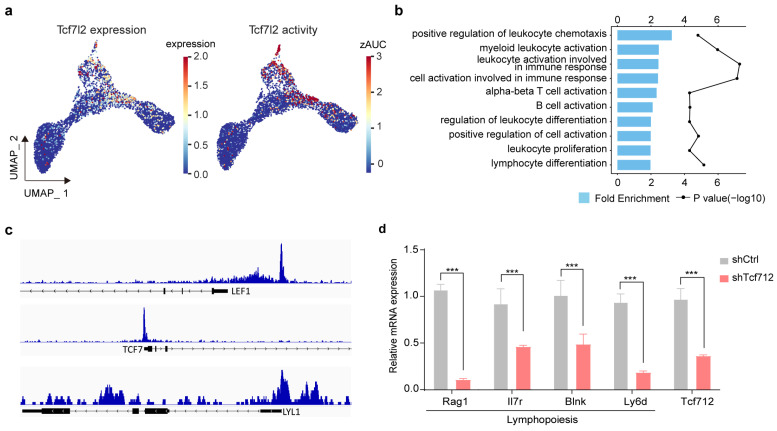
Multi-level validation of *Tcf7l2* function in lymphoid differentiation. (**a**) Expression and activity distribution of *Tcf7l2* in HSPCs. Expression values were log-normalized, and TF activity was calculated using AUC and normalized by z-score. (**b**) Barplot showing the significantly enriched GO terms among TCF7L2 putative targets in human CD34^+^ progenitor cells. (**c**) ChIP-seq demonstrating TCF7L2 binding to the lymphoid gene loci *LEF1*, *TCF7*, and *LYL1*. (**d**) Relative expression of B cell-associated genes and *Tcf7l2* in bone marrow cells following *Tcf7l2* knockdown and culture under B cell differentiation conditions. Statistical analyses were performed using a two-tailed Student’s *t*-test. *N* = 3 biological replicates per group. Data are shown as mean ± standard error of the mean (SEM). *** *p* < 0.001.

To further validate the regulatory role of *Tcf7l2*, we first examined its expression and activity across HSPCs (Figure 3a), which showed preferential enrichment in HSC and lymphoid linage. We then analyzed a publicly available ChIP-seq dataset of human CD34^+^ progenitors [45]. GO enrichment analysis of TCF7L2-bound genes revealed the significant enrichment in lymphocyte-related pathways, including alpha–beta T cell activation, B cell activation, and lymphocyte differentiation (Figure 3b), supporting its involvement in lymphoid lineage specification. In addition, the visualization of ChIP-seq signal tracks demonstrated prominent TCF7L2 binding peaks at the promoters of key lymphoid regulators, including *LEF1*, *TCF7* (*TCF1*) [46], and *LYL1* [47] (Figure 3c), further confirming its direct regulatory effect on lymphoid gene programs. Finally, to functionally assess its role, we performed short hairpin RNA (shRNA)-mediated knockdown of *Tcf7l2* in bone marrow cells, followed by culture under B cell differentiation conditions. Efficient knockdown of *Tcf7l2* was confirmed, and the expression of B cell-associated genes (Rag1, Il7r, Blnk, and Ly6d) was significantly reduced upon *Tcf7l2* knockdown, supporting a functional role for *Tcf7l2* in lymphoid differentiation, particularly in the B cell lineage.

### 2.3. Temporal Dynamics of TF Activity Identifies TFs Contributing to Hematopoietic Lineage Differentiation Biases After Irradiation

**Figure 4:**
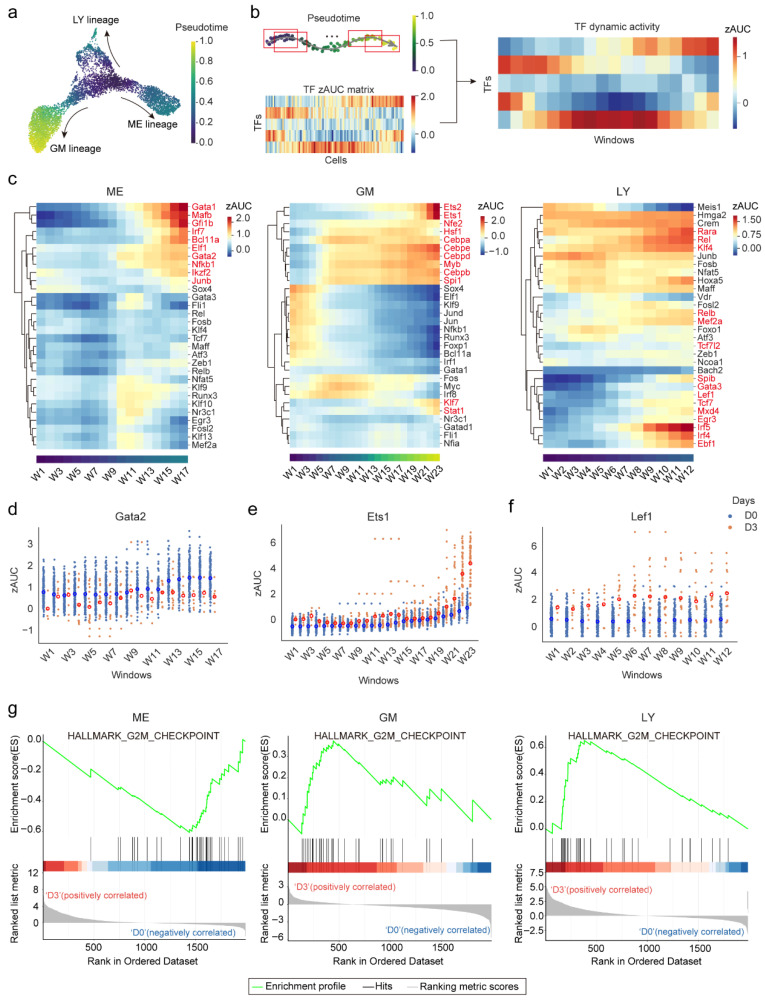
TF dynamic activity analysis explains differentiation bias between D3 and D0. (**a**) UMAP visualization of pseudotime scores along GM, ME, and LY lineages based on our previous pseudotime analysis results [21]. (**b**) Schematic illustration of the window-smoothing strategy applied to zAUC matrix to derive dynamic activity. Red boxes represented windows, and the arrow indicated the direction of differentiation. (**c**) Heatmaps displaying dynamic activity profiles along ME, GM, and LY lineages. TFs with increasing activity along pseudotime were highlighted in red. (**d**–**f**) Jittered scatter plots comparing the dynamic activity of *Gata2* (ME), *Ets2* (GM), and *Lef1* (LY) between D3 and D0. Red circles represented D3, and blue circles represented D0. Red circles represented cells in D3, and blue circles represented cells in D0. The mean values were indicated by brighter-colored circles. (**g**) GSEA showing the reduction in G2M checkpoint activity in ME, but elevation in GM and LY at D3 relative to D0. Red and blue indicate positive and negative correlations with the gene set, respectively.

Having delineated the cell type-specific regulators, we next sought to identify TFs that are correlated with the hematopoietic lineage differentiation progress. To this end, we integrated the single-cell TF activity with pseudotime information from previous analysis [21] along the three major differentiation trajectories—megakaryocyte–erythroid (ME), granulocyte–macrophage (GM), and lymphoid (LY)—to characterize the temporal patterns of lineage-specific TFs (Figure 4a). To reduce the noise inherent in single-cell measurements, we developed a smoothing strategy to estimate the dynamic TF activity profiles within sliding windows, with step sizes constrained by both cell density (defined by cell counts) and pseudotime intervals (Figure 4b). Distinct temporal patterns emerged across the three differentiation lineages (Figure 4c). TF activities that increased along pseudotime included many established lineage regulators, encompassing both factors identified by our regulatory network analysis and additional canonical drivers—such as *Gata1*, *Gata2* [33], *Gfi1b* [34], and *Bcl11a* [48] in the ME lineage; *Spi1* [12], *Cebpa* [49], *Cebpb* [50], *Cebpe* [36], and *Myb* [51] in the GM lineage; and *Irf4* [40], *Irf5* [52], *Ebf1* [53], *Lef1* [46], *Tcf7*(*Tcf1*) [54], and *Spib* [55] in the LY lineage—highlighting these TFs as potential drivers of lineage progression.

**Figure A4:**
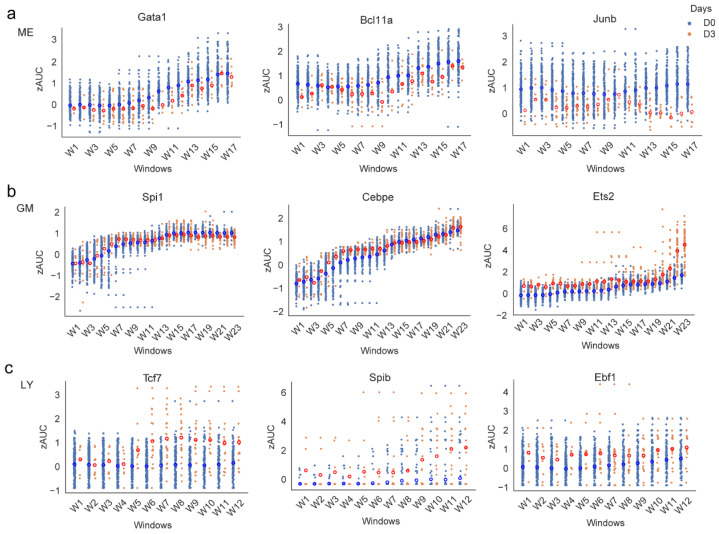
Additional comparison for TF dynamic activity between D3 and D0. (**a**–**c**) Jittered scatter plots comparing the dynamic activity along ME, GM, and LY lineages between D3 and D0. Red circles represented D3, and blue circles represented D0. Red circles represented cells in D3, and blue circles represented cells in D0. The mean values were indicated by brighter-colored circles.

To further identify TFs that are related to hematopoietic lineage differentiation biases following irradiation, we compared the dynamic activity of candidate TFs between pre-irradiation (D0) and post-irradiation (D3). In the ME lineage, erythroid regulators (*Gata1*, *Gata2* [33], and *Bcl11a* [48]), as well as the proliferation-associated factor *Junb* [56], were markedly reduced (Figure 4d and Figure A4a), suggesting impaired erythroid differentiation and diminished proliferative capacity. This is consistent with the observed reduction in this population at D3 post-irradiation (Figure 1c). In the GM lineage, the proliferation-associated factors, *Ets1/2* [57], showed a pronounced increase at the terminal stage, whereas core myeloid regulators (*Spi1* [12] and *Cebpe* [36]) remained largely stable (Figure 4e and Figure A4b), indicating that the increase in proliferation is the main cause for GMP expansion after irradiation. In the LY lineage, T cell regulators (*Lef1*, *Tcf7* [46]) and B cell regulators (*Ebf1* [43], *Spib* [13]) exhibited strong activation (Figure 4f and Figure A4c), suggesting an enhanced differentiation potential following irradiation. Supporting these TF-level changes, pathway-level enrichment analysis of proliferation signatures revealed reduced activity in the ME lineage but increased activity in both the GM and LY lineages (Figure 4g). Collectively, these results indicate that the suppression of both lineage-driving and proliferation-associated TFs underlies the decline in ME differentiation after irradiation, whereas GM expansion is driven primarily by increased proliferation while maintaining stable expression of lineage-specific regulators.

### 2.4. In Silico TF Perturbation Predicts Transcription Factors Regulating Lineage Differentiation After Irradiation

**Figure 5:**
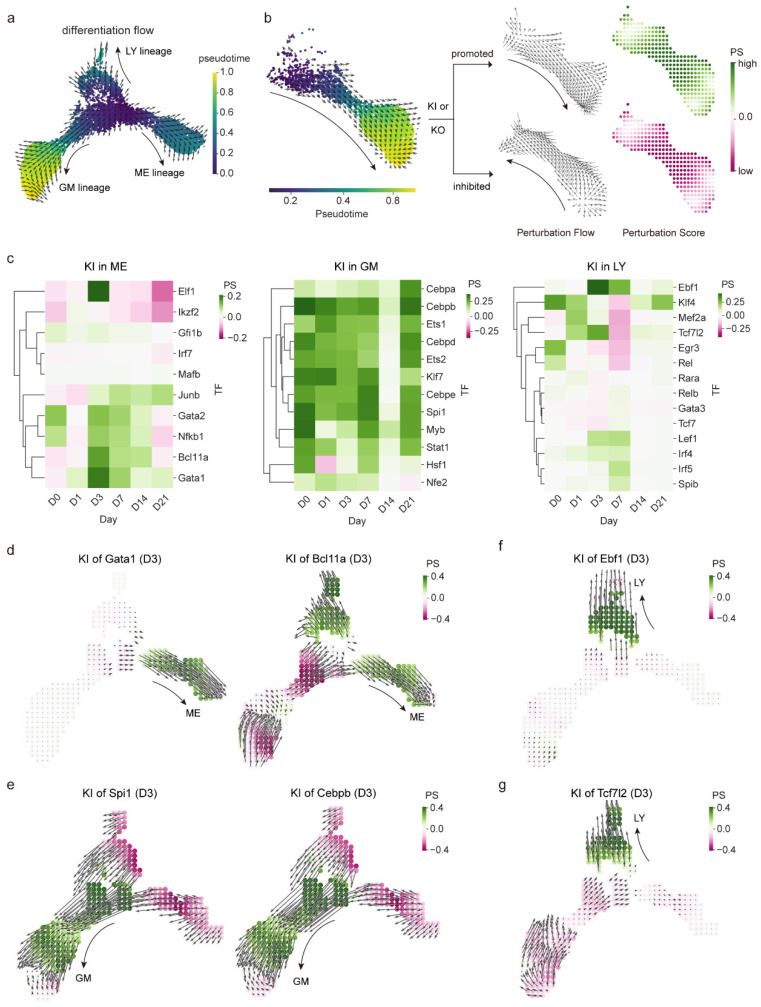
In silico knock-in analysis identifying effects of specific TFs on lineage differentiation. (**a**) UMAP visualization of the differentiation flow along GM, ME, and LY lineages. (**b**) Schematic illustration of TF perturbation modeling using CellOracle. Perturbation effects were represented as perturbation flows and quantified by a perturbation score (PS), defined as the inner product between the perturbation and differentiation flow. Perturbations were simulated as either knock-in (KI) or knock-out (KO). Arrows indicated the direction of differentiation or perturbed differentiation. (**c**) Heatmaps displaying the knock-in effects of specific TFs across ME, GM, and LY lineages at time points following radiation. (**d**–**g**) UMAPs showing the knock-in effects of ME factors (*Gata1* and *Bcl11a*), GM factors *(Spi1* and *Cebpb*), LY factors (*Ebf1* and the novel lymphoid regulator *Tcf7l2*) at D3.

To evaluate the impact of lineage-specific TFs on hematopoietic differentiation, we applied in silico perturbation of TFs using CellOracle [28]. This framework simulates TF loss- or gain-of-function within the inferred regulatory network and estimates how these perturbations redirect cellular differentiation flows along pseudotime (Figure 5a,b). Perturbation-induced deviations were quantified using a perturbation score (PS).

**Figure A5:**
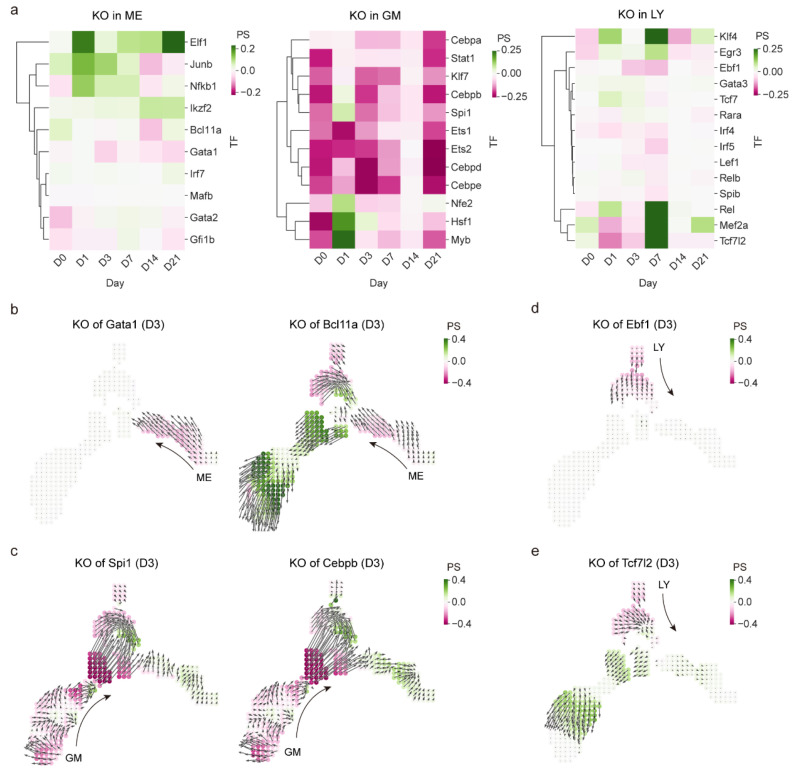
Additional analyses for in silico knock-out perturbations. (**a**) Heatmaps displaying the knock-out effects of specific TFs across ME, GM, and LY lineages at pre- and post-irradiation time points. (**b**–**e**) UMAPs showing the knock-out effects of ME-specific TFs (*Gata1* and *Bcl11a*), GM-specific TFs *(Spi1* and *Cebpb*), and LY-specific TFs (*Ebf1* and the novel lymphoid regulator *Tcf7l2*) at D3. Arrows indicated the direction of perturbed differentiation.

In silico perturbation analysis revealed that knock-in of many lineage-specific TFs could enhance the differentiation of hematopoietic progenitor cells toward their corresponding lineage commitments (Figure 5c, Tables S6–S8). For example, knock-in of erythroid regulators such as *Gata1*, *Gata2*, and *Bcl11a* markedly promoted erythropoiesis beginning at D3. Similarly, knock-in of myeloid drivers (*Spi1* and CEBP family genes) and proliferation-associated factors (*Ets1/2*) consistently enhanced myelopoiesis both before and after radiation. Knock-in of lymphoid regulators, including *Ebf1*, *Lef1*, *Irf4*, *Irf5* and *Spib*, likewise facilitated lymphopoiesis. We also showed the strong pro-differentiation effect of key drivers at D3 (Figure 5d–f). In addition to in silico knock-in analyses, we also performed in silico TF knock-out experiments, which showed that in silico knock-out of specific TFs impaired the differentiation within the corresponding lineages (Figure A5a–d, Tables S6–S8), producing effects opposite to those observed in knock-ins. Together, these results demonstrate the capacity of in silico perturbation modeling to efficiently screen large numbers of candidate regulators and to uncover TF-specific effects on lineage outcomes under irradiation-induced stress.

Our perturbation analysis also supported a role for the newly identified factor *Tcf7l2*, highlighted by our network analysis (Figure 2g). Knock-in of *Tcf7l2* promoted lymphoid differentiation, whereas knock-out impaired it (Figure 5g and Figure A5e), suggesting a previously unappreciated function for *Tcf7l2* in early lymphopoiesis.

**Figure 6:**
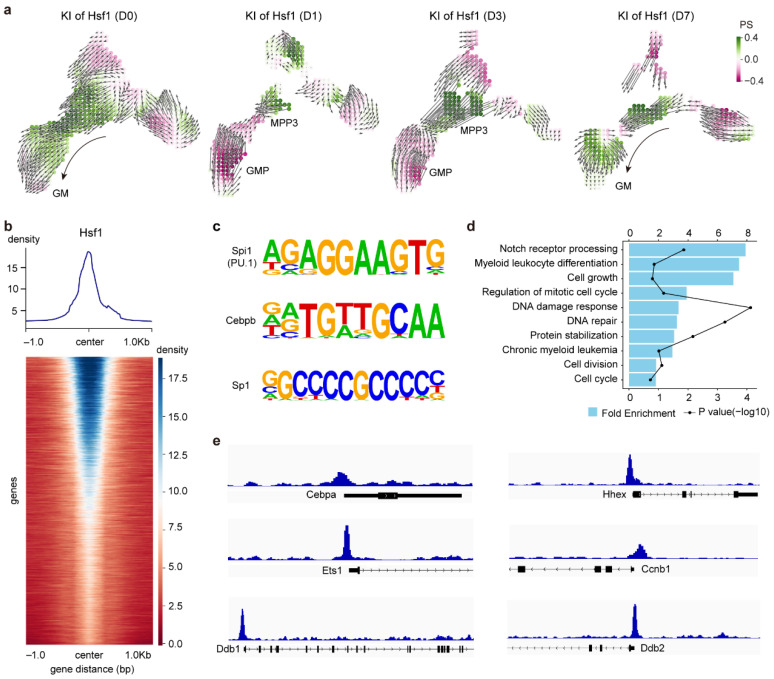
In silico knock-in of *Hsf1* and validation of its effect. (**a**) UMAP showing the in silico knock-in effect of *Hsf1* on GM differentiation across D0, D1, D3, and D7. Arrows indicate the direction of perturbed differentiation. (**b**) Line graph showing average Hsf1 CUT&RUN signal and heatmap showing single-gene Hsf1 occupancy around transcription start sites (TSS ±1 Kb). (**c**) Motif enrichment analysis of Hsf1 CUT&RUN peaks revealing enrichment of motifs corresponding to myeloid regulators (PU.1/Spi1, and Cebpb) and GC-rich zinc finger transcription factors associated with stress-responsive promoters (Sp1). (**d**) Bar plot showing significantly enriched GO terms among Hsf1-bound targets in LSCs (leukemic stem cells). (**e**) CUT&RUN illustrating Hsf1 occupancy at regulatory regions associated with myeloid regulators (*Cebpa* and *Hhex*), cell cycle-related factors (*Ets1* and *Ccnb1*) and DNA damage response-related genes (*Ddb1* and *Ddb2*).

Among the TFs predicted to promote GM differentiation, *Hsf1*, a stress-responsive TF with available pharmacological activators [58,59], was selected for further analysis. In silico perturbation analysis showed that knock-in of *Hsf1* could markedly enhance GM differentiation at D0 and D7, while only promoting MPP3 at D1 and D3 (Figure 6a). To further investigate the role of *Hsf1* in GM differentiation, we leveraged publicly available CUT&RUN data from leukemic stem cells (LSCs) [60], given their reported epigenetic and chromatin accessibility similarities to normal HSCs, particularly at stemness- and stress-related loci [61]. Genome-wide profiling revealed prominent Hsf1 occupancy around transcription start sites (Figure 6b). Motif enrichment analysis further revealed significant enrichment of motifs corresponding to myeloid regulators (PU.1/Spi1 [12] and Cebpb [50]) as well as DNA damage response-related TF Sp1 [62], suggesting that Hsf1 binding occurs within regulatory contexts relevant to myeloid differentiation and stress-responsive transcription. Peak enrichment analysis further indicated that Hsf1-bound genes are involved in Notch receptor processing, myeloid leukocyte differentiation, cell proliferation, and DNA damage response (Figure 6d). Consistent with these pathway-level enrichments, prominent Hsf1 binding signals were observed at key myeloid differentiation regulators (*Cebpa* [49], *Hhex* [63]), cell proliferation regulators (*Ets1* [38], *Ccnb1* [64]), and DNA damage response-related genes (*Ddb1*, *Ddb2* [65]) (Figure 6e). Notably, *Ets1* has been shown above to be a major regulator contributing to GM expansion after irradiation (Figure 4e). Together, these observations suggest that *Hsf1* may participate in transcriptional programs associated with stress responses and myeloid differentiation.

### 2.5. In Vivo Validation of Celastrol Treatment Supports Enhanced Myeloid Recovery After Irradiation

**Figure 7:**
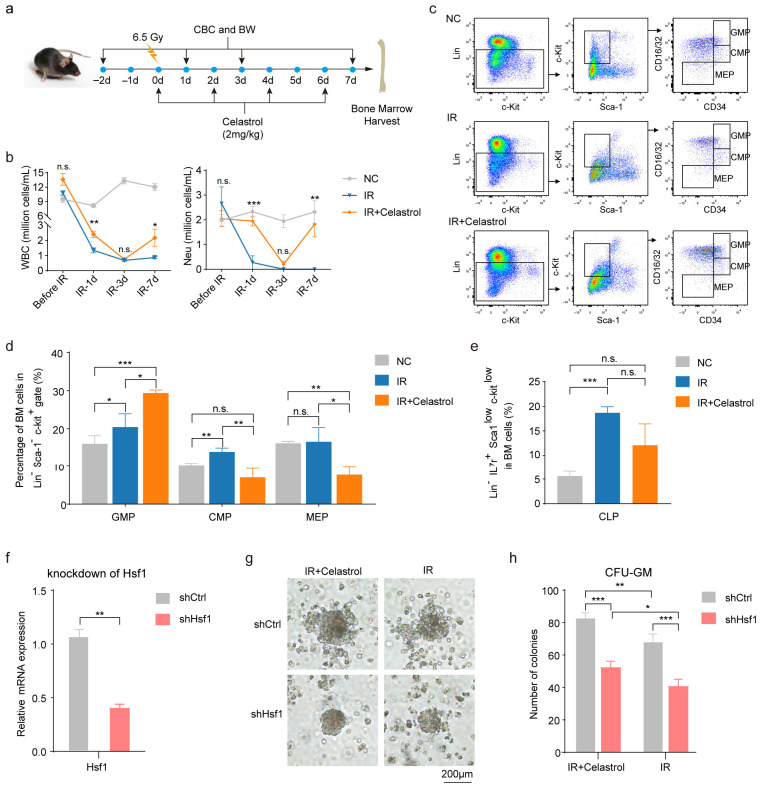
In vivo validation of *Hsf1*-mediated promotion of GM lineage reconstitution after irradiation. (**a**) Experimental design for in vivo *Hsf1* activation. Mice received 6.5 Gy total body irradiation followed by intraperitoneal celastrol administration every 2 days. Complete blood counts (CBCs) and body weight (BW) were measured before irradiation and at post-irradiation (days 2, 4, and 6), and bone marrow was collected at day 7. (**b**) Line plots showing white blood cell (WBC) and neutrophil (Neu) counts before irradiation and across days 1, 3, and 7. Statistical comparisons between IR and IR + celastrol groups were performed using unpaired two-tailed Student’s *t*-test. *N* = 6 mice per group. IR, ionizing radiation; NC, negative control. (**c**) Flow cytometric analysis of bone marrow cells at day 7. *N* = 3 biological replicates per group. (**d**,**e**) Quantification of GMP, CMP, MEP, and CLP percentages in bone marrow at day 7. Statistical analyses were performed using two-way ANOVA followed by Tukey’s multiple comparisons test. *N* = 3 biological replicates per group. (**f**) Relative expression of Hsf1 in shRNA-mediated knockdown (shHsf1) and non-targeting control (shCtrl). (**g**,**h**) A representative colony in each group and the quantification of colony numbers in colony-forming unit–granulocyte–macrophage (CFU–GM) assay. Statistical analyses were performed using unpaired two-tailed Student’s *t*-test. *N* = 3 mice per group. Data are shown as mean ± SEM. * *p* < 0.05, ** *p* < 0.01, *** *p* < 0.001, n.s., not significant.

**Figure A6:**
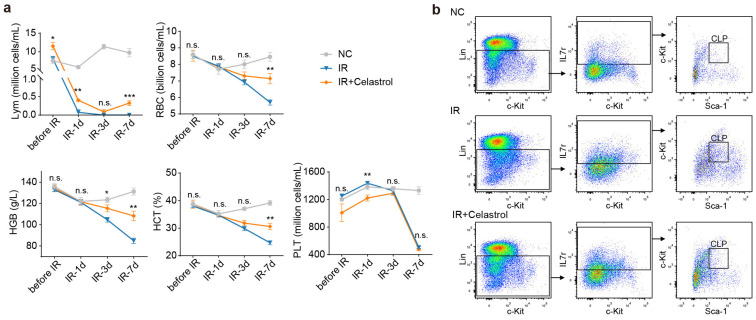
Additional results for in vivo *Hsf1* activation experiment. (**a**) Line plots showing the complete blood counts including lymphocytes (Lym), red blood cell (RBC) and platelet (PLT) counts, as well as hemoglobin (HGB) and hematocrit (HCT) concentration before irradiation and at days 1, 3, and 7. Statistical comparisons between IR and IR + celastrol groups were performed using unpaired two-tailed Student’s *t*-test. *N* = 6 mice per group. IR, ionizing radiation; NC, negative control. (**b**) Flow cytometric analysis of bone marrow lymphoid progenitor compartments at day 7. *N* = 3 biological replicates per group. Data are shown as mean ± SEM. * *p* < 0.05, ** *p* < 0.01, *** *p* < 0.001, n.s., not significant.

To functionally verify whether activation of *Hsf1* could promote GM differentiation after irradiation, we conducted in vivo post-irradiation dosing experiments using celastrol, a small-molecule activator known for enhancing *Hsf1* function [58] (Figure 7a). The mice were assigned to three groups: control, IR, and celastrol-IR. The mice in both IR and celastrol-IR groups received 6.5 Gy total body irradiation, and mice in celastrol-IR group were intraperitoneal-injected with celastrol 30 min after irradiation, followed by additional doses at D2, D4, and D6. Peripheral blood analysis showed that white blood cells (WBCs) and neutrophils declined to their nadir at day 3 after irradiation in both irradiated groups (Figure 7b). However, by day 7, celastrol-treated mice exhibited a robust rebound in both parameters, with neutrophil counts close to non-irradiated baseline levels, whereas vehicle-treated mice displayed minimal recovery (Figure 7b). Lymphoid- and erythroid-related indices showed only modest improvement by day 7 (Figure A6a). Flow cytometric analysis of bone marrow hematopoietic progenitor cells at day 7 post-irradiation (Figure 7c and Figure A6b) further revealed a significant increase in GMP fractions in celastrol-treated mice, accompanied by reduced CMP and MEP, while the CLP was not significantly affected (Figure 7d,e). These in vivo observations align closely with the in silico knock-in predictions above, which forecast enhanced GM output upon *Hsf1* activation (Figure 6a).

To further directly assess the role of *Hsf1* in myeloid differentiation, we performed shRNA-mediated knockdown of *Hsf1* in bone marrow cells at day 7 post-irradiation, followed by a colony-forming unit–granulocyte–macrophage (CFU–GM) assay. *Hsf1* expression was effectively reduced in the shHsf1 group, confirming the knockdown efficiency (Figure 7f). In the CFU–GM assay, *Hsf1* knockdown significantly decreased the number of CFU–GM colonies in both the IR and IR + celastrol groups (Figure 7g,h), indicating that *Hsf1* plays a necessary role in post-irradiation myeloid differentiation. In the absence of *Hsf1* knockdown, the IR + celastrol group exhibited a significantly higher number of CFU–GM colonies compared to the IR group (Figure 7h), further supporting the promotive effect of celastrol on myeloid differentiation and proliferation following irradiation. In contrast, under *Hsf1* knockdown condition, although the IR + celastrol group still showed higher colony numbers than the IR group, the overall levels were markedly reduced compared to their non-knockdown counterparts (Figure 7h), indicating that the promotive effect of celastrol on post-irradiation myeloid differentiation is substantially attenuated upon *Hsf1* suppression.

Together, these results demonstrated that pharmacological activation of *Hsf1* promotes early myeloid reconstitution following irradiation. Combined with the knockdown and CFU–GM assay, our findings further establish a functional requirement for *Hsf1* in post-irradiation myeloid differentiation and show that the promotive effects of celastrol are substantially attenuated upon *Hsf1* suppression. These results highlight the potential of *Hsf1*-targeted strategies to enhance hematopoietic recovery after irradiation.

## 3. Discussion

HSPCs are regulated by complex gene regulatory networks that orchestrate their self-renewal and lineage commitment. Despite extensive knowledge of individual TFs in steady-state hematopoiesis, the mechanisms by which high-dose irradiation rewires these transcriptional regulatory programs and whether perturbing key TFs can facilitate hematopoietic recovery by redirecting lineage trajectories remain poorly understood. Here, using scRNA-seq profiles from irradiated and control samples, we delineated the dynamic remodeling of HSPC population states and performed population-level regulatory network analysis. By analyzing the dynamics of TF activity along hematopoietic lineages, we identified lineage-specific candidate drivers and applied in silico perturbation to predict their regulatory effects on differentiation. Notably, we identify *Tcf7l2* as a previously unrecognized regulator associated with lymphoid differentiation, and highlight the therapeutic potential of *Hsf1* activation in promoting myeloid differentiation following irradiation.

Previous studies have described multiple isolated aspects of HSPCs response to irradiation, including HSC exhaustion [2], loss of erythroid progenitors [66], enhanced lymphoid differentiation [67], and long-term myeloid skewing [68,69]. However, these observations remain fragmented and lack an integrated view across the entire compartment. By profiling all HSPC subpopulations over time, we uncovered a coordinated but phase-specific remodeling: both HSCs and MPPs were depleted at D1, accompanied by transient expansions of MEP, GMP, and CLP. From D3 onward, MEP and CLP pools contracted sharply, whereas GMP continued to expand, with the more mature CLP subset uniquely maintained. Consistent with these population shifts, activity dynamics and enrichment analyses revealed suppressed proliferation and differentiation programs in MEP at D3, contrasted with heightened proliferative activity in GMP and CLP and increased differentiation activity within CLP. Collectively, these analyses provide a unified, lineage-resolved view of how HSPC subpopulations undergo fate remodeling during the acute response to irradiation.

Transcriptional regulation of HSPC fate has been shaped by a set of canonical lineage-specifying TFs, including *Gata1* [33], *Spi1* [12], and *Ebf1* [43]. Through reconstruction of the lymphoid regulatory network, we uncovered *Tcf7l2* as an unexpected but strongly connected lymphoid regulator, tightly linked to established factors such as *Irf4*, *Ebf1*, and *Klf4*. Although *Tcf7l2* has been primarily studied as a mediator of Wnt/β-catenin signaling, which plays important roles in hematopoietic and immune regulation [70,71], its specific involvement in lymphoid lineage specification has been less clearly defined. Here, converging evidence supports its involvement in lymphoid lineage specification. *Tcf7l2* showed preferential expression and activity in HSC and lymphoid-biased progenitors, and ChIP-seq analysis revealed binding to key lymphoid regulators, including *LEF1*, *TCF7*, and *LYL1*. Consistently, in silico perturbation predicted that *Tcf7l2* knock-in promotes lymphoid differentiation in D3, while knockdown reduced the expression of B cell-associated genes during differentiation. Together, these findings suggest that *Tcf7l2* contributes to the establishment of lymphoid gene programs during hematopoietic differentiation.

As a promising strategy for predicting TF or compound functions, computational perturbation modeling has gained increasing attention due to its substantial potential. Integrating in silico prediction with in vivo validation provides a powerful framework for uncovering regulatory mechanisms and identifying candidates with translational relevance. Recent studies have demonstrated the effectiveness of this approach. For example, Kamimoto et al. applied in silico TF perturbation to identify regulators of axial mesoderm differentiation, which were subsequently validated using CRISPR-based perturbations [28]. Similarly, Macias et al. developed a drug discovery platform to predict small-molecule interventions for thrombocytopenia and validated their effects on megakaryocyte differentiation in vivo [72].

In this study, we established a similar prediction-to-validation framework. We first reconstructed the transcriptional regulatory landscape of hematopoietic recovery following irradiation and identified candidate TFs through dynamic TF activity analysis across hematopoietic lineages. We then performed in silico knock-in and knock-out simulations for these candidate regulators across multiple time points before and after irradiation. Notably, the predicted perturbation effects recapitulated the known functions of several established lineage regulators. For example, *Gata1* and *Gata2* promoted ME differentiation, *Spi1* and *Cebpb* enhanced GM differentiation, and *Ebf1* and *Lef1* promoted LY differentiation. These findings demonstrate the utility of computational perturbation modeling for identifying lineage-regulating TFs and suggest potential targets for promoting hematopoietic recovery following irradiation.

Among the predicted regulators, we further focused on *Hsf1*, a factor that can be pharmacologically activated by celastrol [59,73]. *Hsf1* has previously been implicated in maintaining HSC function under aging-associated stress [74,75] and sustaining leukemia stem cell self-renewal in AML [60]. Our perturbation analysis revealed a context-dependent role for *Hsf1*: the activation of *Hsf1* promoted GM differentiation at D0 and D7 but exhibited opposing effects at D1 and D3 by enhancing MPP3 while suppressing GMP. Given that *Hsf1* is known to induce G2 cell cycle arrest and facilitate DNA repair following radiation [76,77], this transient suppression of GMP may serve as a protective mechanism during the early post-irradiation phase. Consistently, in vivo pharmacological activation of *Hsf1* by celastrol promoted GMP expansion and accelerated the recovery of white blood cell counts by D7. Moreover, shRNA-mediated knockdown of *Hsf1* significantly reduced CFU–GM colony formation under both basal and celastrol-treated conditions, demonstrating a functional requirement for *Hsf1* in post-irradiation myeloid differentiation and suggesting that *Hsf1* contributes to the promotive effects of celastrol. Together, these results support a role for *Hsf1* in driving myeloid-biased hematopoietic recovery following radiation injury. In contrast to TF-targeted protective strategies such as NRF2, which broadly supports HSPCs self-renewal and proliferation [22], and NF-κB, which regulates HSC activation and self-renewal through interactions with bone marrow microenvironment [23], *Hsf1* preferentially promotes myeloid expansion, highlighting a lineage-biased regulation mechanism.

Our study provides an integrated and temporally resolved framework for understanding how high-dose irradiation reshapes transcriptional regulation and lineage trajectories in the HSPC compartment. By combining single-cell transcriptomics, population-level regulatory network analysis, and computational perturbation modeling, we systematically identified lineage-specific regulatory drivers and demonstrated that their functions can be inferred and predicted following irradiation. The discovery of *Tcf7l2* as a previously unrecognized lymphoid regulator and the in vivo validation of *Hsf1* highlight the power of this integrative strategy to not only uncover canonical regulators but also reveal context-dependent regulators. These results not only deepen our understanding of stress-induced hematopoietic regulation but also underscore the potential of TF-directed interventions to improve bone marrow recovery following cytotoxic injury.

## 4. Materials and Methods

### 4.1. Mice

Six to eight-week-old C57BL/6 mice were purchased from the Vital River Laboratory Animal Technology (Beijing, China). All mice were kept in a specific pathogen-free (SPF) barrier environment and were continuously provided with sterilized food, water, and bedding.

### 4.2. Radiation and Treatment

To establish an in vivo model of radiation-induced bone marrow (BM) injury, mice were subjected to 6.5 Gy total body irradiation using a ^60^Co gamma ray source at a dose rate of 69 cGy/min at the Beijing Institute of Radiation Medicine (Beijing, China), as previously described [21,78,79]. To evaluate the radioprotective potential of celastrol, the mice received intraperitoneal injections of celastrol (2 mg/kg, #S1290; Selleck, Houston, TX, USA) or vehicle control 30 min post-irradiation, followed by additional doses administered at 2-day intervals, based on previously reported in vivo studies [80,81,82]. Celastrol was prepared in a vehicle consisting of 5% DMSO and 95% corn oil. Body weights and hematological parameters were assessed at baseline (2 days before irradiation) and on days 1, 3, and 7 after irradiation.

### 4.3. Hematological Analyses

Approximately 20 µL of peripheral whole blood was collected from the tail vein of mice for hematological analysis without euthanasia. Complete blood counts, including white blood cell (WBC), lymphocyte (Lym), neutrophil (Neu), red blood cell (RBC), and platelet (PLT) counts, as well as hemoglobin (HGB) concentration, were measured using an automated Celltas ES Hematology Analyzer (Nihon Kohden, Tokyo, Japan).

### 4.4. Flow Cytometric Analyses

Whole bone marrow (BM) was isolated by flushing the hindlimb bones (femurs and tibias) with PBS. The BM single-cell suspension was filtered through a 100 μm cell strainer to remove debris. Red blood cells were lysed on ice using RBC lysis solution (R1010; Solarbio, Beijing, China), and the remaining cells were washed with PBS. The cells were then stained with the fluorophore-conjugated antibodies targeting the following antigens: anti-Lineage Cocktail (92-7770-T100; Tonbo Biosciences, San Diego, CA, USA), anti-Sca-1 (562729; BD Biosciences, Franklin Lakes, NJ, USA), anti-c-Kit (60-1172-U100; Tonbo Biosciences), anti-CD16/32 (17-0161-82; eBioscience, San Diego, CA, USA), anti-CD34 (551387; BD Biosciences), and anti-IL7r (20-1271-U100; TONBO). Antibase conjugates included FITC, BV421, PE-Cy7, APC, and PE. After incubation for 30 min at 4 °C in the dark, the cells were washed and resuspended in cell staining buffer (420201; BioLegend, San Diego, CA, USA). The samples were analyzed using a BD FACSCanto™ II flow cytometer (BD Biosciences). The data were analyzed by the FlowJo software (v10.0; BD Biosciences). The results are the mean of three independent experiments, each performed in triplicate.

### 4.5. scRNA-seq

For additional scRNA-seq dataset, the enriched Lin- BM cells from non-irradiated and irradiated mice were converted to barcoded scRNA-seq libraries using the Chromium Single Cell 3′ Library, Gel Bead & Multiplex Kit, and Chip Kit (10× Genomics, Pleasanton, CA, USA) following the manufacturer’s instructions, aiming for an estimated 10,000 cells per library. The samples were processed using kits pertaining to V3.1 barcoding chemistry of 10× Genomics. Single samples are always processed in a single well of a PCR plate, allowing all cells from a sample to be treated with the same master mix and in the same reaction vessel. All samples were processed in parallel in the same thermal cycler. The generated scRNA-seq libraries were sequenced on a NovaSeq sequencer (Illumina, San Diego, CA, USA) in CapitalBio (Beijing, China).

### 4.6. Gene Expression Quantification, Quality Control, and Batch Correction for scRNA-seq

The Cell Ranger software (version 4.0.0; 10× Genomics) was used for sample demultiplexing, barcode processing, and single-cell 3′ counting. The fastq files for each sample were processed with the count function in Cell Ranger, which was used to align the reads to mouse genome (build mm10) and quantify the gene expression levels in single cells. To filter out low-quality cells for each sample, the cells that had either fewer than 500 or over 5000 expressed genes were removed. To filter out dead or dying cells, the cells that had over 20% unique molecular identifiers (UMIs) derived from mitochondrial genome were further removed. Gene expression in single cells was normalized using SCTransform function in R package Seurat (v4.0) [83], and the anchor-based batch correction method was employed to merge samples from different groups.

### 4.7. Cells Clustering and Hematopoietic Cell Clusters Annotation of scRNA-seq

Principal component analysis (PCA) was performed for dimensionality reduction and 30 principal components (PCs) were then used for further analysis. UMAP visualization was performed to embed the neighborhood graph and display the topology of the data. For cell clustering, we used the FindClusters function in Seurat (v4.0), which implements a shared nearest neighbor (SNN) modularity optimization-based clustering algorithm. The Scrublet package [84] was used to identify doublets (two cells encapsulated in a single droplet) from scRNA-seq data with default parameters. Cell clusters with extremely low nUMI count and high proportion of doublets were assigned as low-quality clusters and were excluded from further analyses. The clusters annotation was performed using a set of previously reported canonical marker genes of hematopoietic stem and progenitor cells (HSPCs) (Figure A2a).

### 4.8. Relative Abundance Analysis

MELD analysis (version 1.0.2) [30] was used to estimate the likelihood of each cell across time points. Relative likelihood values were obtained by comparing post-irradiation samples with the pre-irradiation baseline. Based on the resulting relative likelihood values, the cells were classified into up, no significance (N.S.), and down response modes using K-means clustering, with thresholds of <0.45 and >0.55 defining down and up responses, respectively.

### 4.9. Gene Set Variation Analysis

Gene set variation analysis (GSVA) was performed using the GSVA R package (version 2.4.0) [85] to estimate pathway activity at the single-cell level. The expression data were log-normalized prior to analysis, and the GSVA scores were computed for each cell. The gene sets were obtained from the MSigDB Hallmark collection (Mus musculus) [86]. The GSVA scores were calculated using the default parameters. The resulting GSVA scores were averaged at the population level across pre- and post-radiation time points and then z-score normalized across time points for each population.

### 4.10. TF Activity Analysis and Cell Type-Specific Network Analysis

SCENIC analysis was performed to infer the regulatory networks and TF activity, with the pySCENIC package (version 0.12.1) [32]. Co-expression modules were inferred from single-cell transcriptomes based on Mus musculus ranking databases (mm10_10kb_up_10kb_down and mm10_500bp_up_100bp_down). Regulons were defined using the allTFs_mm TF list and motifs-v10nr annotations, and their activity scores were quantified through enrichment analysis in each cell. These datasets are downloaded from the cistargetDBs website: https://resources.aertslab.org/cistarget/ (accessed on 20 November 2025).

The top 10 cell type-specific regulons were selected according to their regulon specificity scores (RSSs) [32], which quantify the association between regulon activity and cell identity. Key TF–target interactions within each regulon were further filtered using Gaussian Mixture Models (GMMs) to retain the most significant regulatory edges. The resulting regulons and their high-confidence interactions were then integrated into cell type-specific regulatory networks and visualized using Cytoscape (version 3.10.4) [87].

### 4.11. Dynamic Activity Calculation

To characterize the dynamic changes in TF activity along each lineage while minimizing noise, we applied a sliding window-smoothing strategy. The cells were first ordered by pseudotime and partitioned into windows of 300 cells. The step size was initially defined as the default cell spacing (100 cells) and then adjusted to ensure that the corresponding pseudotime distance fell within a predefined interval (0.03–0.06). If the pseudotime distance did not meet this criterion, the step size was recalculated using the interval thresholds to prevent abrupt or overly small fluctuations in activity between adjacent windows. The pseudotime values were normalized to the range (0, 1) using min–max scaling. The mean TF activity within each window was then calculated sequentially to obtain smoothed dynamic activity.

Lineage-specific TFs were defined as the top 30 TFs ranked by maximum RSS in cell types of each lineage (ME: MPP2 and MEP; GM: MPP3 and GMP; and LY: MPP4 and CLP).

### 4.12. In Silico TF Perturbation Analysis

In silico TF perturbation was performed using CellOracle (version 0.20.0) [28]. Knock-out (KO) or knock-in (KI) of single TF was simulated within the inferred regulatory networks to assess their influence on target genes and subsequent changes in cell identity. For knock-in simulations, the expression of the perturbed TF was set to the greater of 1 or twice the 95th percentile of its observed expression. The resulting perturbed flow representing the direction of differentiation following TF perturbation, whereas the developmental flow was derived from pseudotime along the lineage trajectory. The effect of perturbation on differentiation was quantified by the perturbation score (PS), defined as the inner product of the perturbed and developmental flow vectors. A higher PS indicates that the perturbation promotes differentiation along the lineage direction, whereas a lower or negative PS reflects repression or the reversal of differentiation progression. The PS along each lineage was averaged to quantify the overall perturbation effect on that lineage.

### 4.13. CUT&RUN Data Analysis and Target Gene Identification

Reads were aligned to the mm10 reference genome using Bowtie2 (v2.5.4) [88] with default parameters. High-confidence Hsf1 binding peaks were called using MACS3 (v3.0.3) [89] with default parameters. The peaks were subsequently annotated to the nearest transcription start site (TSS) and genomic regions using the tool annotatePeaks.pl in HOMER (v5.1) [90], allowing the assignment of putative target genes based on promoter-proximal or gene body peaks. Motif enrichment analysis was performed using findMotifsGenome.pl in HOMER (v5.1), with matched background regions to identify significantly enriched transcription factor binding motifs. Normalized coverage tracks (bigWig files) were generated from the deduplicated BAM files using tool bamCoverage in deepTools (v3.5.6) [91] for the visualization in genome browsers.

### 4.14. ChIP-seq/CUT&RUN Data Visualization and Gene Enrichment Analysis

BigWig files from the Hsf1 (mouse, mm10) and TCF7L2 (human, hg38) experiments were loaded into the Integrative Genomics Viewer (IGV, version 2.19.6) [92]. Signal tracks were visualized to examine peak enrichment at the promoters of representative lineage-specific or function-specific genes, allowing the assessment of Hsf1 or TCF7L2 binding patterns. Putative target genes were subjected to gene ontology (GO) enrichment analysis using the clusterProfiler R package (version 4.18.0) [93]. The genes were annotated to GO biological process terms, and pathways with an adjusted *p* < 0.05 were considered to be significantly enriched.

### 4.15. Gene Set Enrichment Analysis

Gene set enrichment analysis (GSEA) was performed using the clusterProfiler R package (version 4.18.0) [93]. The genes were ranked by their average log2 fold change within each lineage, and enrichment was evaluated against the MSigDB Hallmark gene set, HALLMARK_G2M_CHECKPOINT. The gene set in each lineage with an enrichment score (ES) > 0 and a false discovery rate (FDR) < 0.05 were defined as upregulated modules, while those with ES < 0 and FDR < 0.05 were defined as downregulated modules.

### 4.16. Lentiviral Transduction and Flow Cytometric Sorting of Tcf7l2- and Hsf1-Knockdown Bone Marrow Cells

Mouse bone marrow cells were transduced with lentiviral vectors expressing GFP-tagged shRNAs targeting *Tcf7l2* (shTcf7l2), targeting *Hsf1* (shHsf1), or a non-targeting control (shCtrl). For *Hsf1* knockdown experiments, bone marrow cells were isolated from mice at day 7 post-irradiation, with or without celastrol treatment. The cells were cultured in the RPMI-1640 medium supplemented with 10% fetal bovine serum (FBS) (SPC500V; ScienProCell, Beijing, China), 1% penicillin–streptomycin (Pen-Strep) (VCM3004, VIVICUM, Beijing, China), 2 mM L-glutamine (G7513; Sigma-Aldrich, St. Louis, MO, USA), 50 μM β-mercaptoethanol (β-ME) (M3148; Sigma-Aldrich), stem cell factor (SCF, 100 ng/mL) (250-03-10UG; Peprotech, Cranbury, NJ, USA), thrombopoietin (TPO, 50 ng/mL) (315-14-10UG; Peprotech), Flt3 ligand (Flt3-L, 100 ng/mL) (250-31L-10UG; Peprotech), and interleukin-6 (IL-6, 20 ng/mL) (216-16-10UG; Peprotech). After 72 h of transduction, GFP-positive cells were sorted by flow cytometry for downstream analyses. Each condition was performed in triplicate. The sequences of shTcf7l2, shHsf1, and shCtrl are listed in Table S9.

### 4.17. B Cell Differentiation Assay

Flow cytometry-sorted, stably knockdown bone marrow cells were cultured for B cell differentiation in the X-VIVO 15 medium (04-418Q; Lonza, Basel, Switzerland) supplemented with 1% bovine serum albumin (BSA) (A8010; Solarbio), 0.5% Pen-Strep, 2 mM L-glutamine, 50 μM β-ME, interleukin-7 (IL-7, 20 ng/mL) (217-17-10UG; Peprotech), SCF (40 ng/mL), and Flt3-L (100 ng/mL). The cells were cultured under these conditions for 3 days prior to downstream analyses. The experimental protocol was established with reference to the methods reported by Yang et al. [94].

### 4.18. RNA Extraction and RT-qPCR

Total RNA was extracted from bone marrow cells after 3 days of differentiation using the TRIzol reagent (15596018CN; Thermo Fisher, Waltham, MA, USA) according to the manufacturer’s instructions. RNA concentration and purity were determined prior to downstream analyses.

Complementary DNA (cDNA) was synthesized using a reverse transcription kit (RR037A; TaKaRa, Kusatsu, Shiga, Japan). Quantitative real-time PCR (qRT–PCR) was performed using the KAPA SYBR FAST Universal qPCR Kit (KK4601; KAPA Biosystems, Wilmington, MA, USA) to measure the expression levels of target genes. Primer sequences for B-lymphopoiesis-related genes [95], as well as for *Tcf7l2* and *Hsf1*, are listed in Table S10.

### 4.19. CFU–GM Colony Formation Assay

Bone marrow cells were seeded at a density of 2 × 10^4^ cells per mL in methylcellulose-based medium (M3534; STEMCELL, Vancouver, BC, Canada). Each condition was plated in triplicate. After 7 days of incubation, CFU–GM colonies were identified and counted under a light microscope (Nikon, Tokyo, Japan).

### 4.20. Statistical Analyses

Statistical analyses were performed using GraphPad Prism (version 10.6.1). The data are shown as mean ± standard error of the mean (SEM). Statistical significance was determined through two-way ANOVA with Tukey’s multiple comparisons test or the multiple *t*-test. *p* < 0.05 was considered to be statistically significant. All experiments were repeated three or six times independently. Hematological analyses were performed in six independent replicates, and flow cytometry analyses and knockdown analyses were performed in three independent replicates.

## Data Availability

The mouse HSPC scRNA-seq dataset [21] is available in the Genome Sequence Archive (GSA accession: CRA018447) [96]. The raw sequence data reported in this paper have been deposited in GSA (GSA accession: CRA035475). A ChIP-seq dataset profiling TCF7L2 binding in human CD34^+^ progenitor cells (GEO accession: GSM7224402) [45] was used in this study. Corresponding bigWig peak call tracks (q < 1 × 10^−10^) were obtained from the ChIP-Atlas dataset (SRX097075) [97]. Putative TCF7L2 target genes were retrived from the Cistrome Data Browser (CistromeDB ID: 8418) [98]. In addition, a publicly available CUT&RUN dataset profiling Hsf1 binding in leukemia stem cell (SRA accession: SRR17008033) was used [60].

## Supplementary Materials

The following supporting information can be downloaded at: https://www.mdpi.com/article/10.3390/ijms27083522/s1.

## Abbreviations

The following abbreviations are used in this manuscript:

HSPCs	hematopoietic stem and progenitor cells
HSCs	hematopoietic stem cells
MPPs	multipotent progenitors
BM	directory of open access journals
TF	transcription factor
H-ARS	hematopoietic acute radiation syndrome
G-CSF	granulocyte colony-stimulating factor
TPO	thrombopoietin
MCS	mesenchymal stromal cell
GRNs	gene regulatory networks
scRNA-seq	single-cell RNA sequencing
NRF2	nuclear factor erythroid 2-related factor 2
GMP	granulocyte–macrophage progenitor
CLP	common lymphoid progenitor
MEP	megakaryocyte and erythroid progenitor
GM	granulocyte–macrophage lineage
ME	megakaryocyte–erythroid lineage
LY	lymphocyte lineage
N.S.	no significance
UMAP	uniform manifold approximation and projection
GSVA	gene set variation analysis
RSS	regulon specificity score
GMMs	Gaussian Mixture Models
LSC	leukemic stem cells
GO	gene ontology
PS	perturbation score
KO	knock-out
KI	knock-in
WBC	white blood cell
Neu	neutrophil
Lym	lymphocyte
HGB	hemoglobin
HCT	hematocrit
PLT	platelet
RBC	red blood cell
TSS	transcription start site
SEM	standard error of the mean
shRNA	short hairpin RNA
shHsf1	short hairpin RNA targeting Hsf1
shTcf7l2	short hairpin RNA targeting Tcf7l2
shCtrl	control shRNA
CFU–GM	colony-forming unit–granulocyte–macrophage

## References

[1] Shao L., Luo Y., Zhou D. (2014). Hematopoietic Stem Cell Injury Induced by Ionizing Radiation. Antioxid. Redox Signal..

[2] Zhang Y., Chen X., Wang X., Chen J., Du C., Wang J., Liao W. (2024). Insights into Ionizing Radiation-Induced Bone Marrow Hematopoietic Stem Cell Injury. Stem Cell Res. Ther..

[3] Orschell C.M., Wu T., Patterson A.M. (2022). Impact of Age, Sex, and Genetic Diversity in Murine Models of the Hematopoietic Acute Radiation Syndrome (H-ARS) and the Delayed Effects of Acute Radiation Exposure (DEARE). Curr. Stem Cell Rep..

[4] Hofer M., Pospíšil M., Komůrková D., Hoferová Z. (2014). Granulocyte Colony-Stimulating Factor in the Treatment of Acute Radiation Syndrome: A Concise Review. Molecules.

[5] Du C., Chen J., Wang J. (2025). New Insights into the Generation and Function of Megakaryocytes in Health and Disease. Haematologica.

[6] Diaz M.F., Horton P.D., Dumbali S.P., Kumar A., Livingston M., Skibber M.A., Mohammadalipour A., Gill B.S., Zhang S., Cox C.S. (2020). Bone Marrow Stromal Cell Therapy Improves Survival after Radiation Injury but Does Not Restore Endogenous Hematopoiesis. Sci. Rep..

[7] Rezvani M. (2021). Therapeutic Potential of Mesenchymal Stromal Cells and Extracellular Vesicles in the Treatment of Radiation Lesions—A Review. Cells.

[8] Friedman A.D. (2009). Cell Cycle and Developmental Control of Hematopoiesis by Runx1. J. Cell. Physiol..

[9] Porcher C., Chagraoui H., Kristiansen M.S. (2017). SCL/TAL1: A Multifaceted Regulator from Blood Development to Disease. Blood.

[10] Jiang M., Xu S., Bai M., Zhang A. (2021). The Emerging Role of MEIS1 in Cell Proliferation and Differentiation. Am. J. Physiol.-Cell Physiol..

[11] Ferreira R., Ohneda K., Yamamoto M., Philipsen S. (2005). GATA1 Function, a Paradigm for Transcription Factors in Hematopoiesis. Mol. Cell. Biol..

[12] Zakrzewska A., Cui C., Stockhammer O.W., Benard E.L., Spaink H.P., Meijer A.H. (2010). Macrophage-Specific Gene Functions in Spi1-Directed Innate Immunity. Blood.

[13] Tangye S.G. (2025). SPI-Ing on Human B-Cell Development. Blood.

[14] Theilgaard-Mönch K., Pundhir S., Reckzeh K., Su J., Tapia M., Furtwängler B., Jendholm J., Jakobsen J.S., Hasemann M.S., Knudsen K.J. (2022). Transcription Factor-Driven Coordination of Cell Cycle Exit and Lineage-Specification in Vivo during Granulocytic Differentiation. Nat. Commun..

[15] Muench D.E., Olsson A., Ferchen K., Pham G., Serafin R.A., Chutipongtanate S., Dwivedi P., Song B., Hay S., Chetal K. (2020). Mouse Models of Neutropenia Reveal Progenitor-Stage-Specific Defects. Nature.

[16] Shan Q., Li X., Chen X., Zeng Z., Zhu S., Gai K., Peng W., Xue H.-H. (2021). Tcf1 and Lef1 Provide Constant Supervision to Mature CD8^+^ T Cell Identity and Function by Organizing Genomic Architecture. Nat. Commun..

[17] Sigvardsson M. (2023). Transcription Factor Networks Link B-Lymphocyte Development and Malignant Transformation in Leukemia. Genes Dev..

[18] Liggett L.A., Sankaran V.G. (2020). Unraveling Hematopoiesis Through the Lens of Genomics. Cell.

[19] Buenrostro J.D., Corces M.R., Lareau C.A., Wu B., Schep A.N., Aryee M.J., Majeti R., Chang H.Y., Greenleaf W.J. (2018). Integrated Single-Cell Analysis Maps the Continuous Regulatory Landscape of Human Hematopoietic Differentiation. Cell.

[20] Gao P., Chen C., Howell E.D., Li Y., Tober J., Uzun Y., He B., Gao L., Zhu Q., Siekmann A.F. (2020). Transcriptional Regulatory Network Controlling the Ontogeny of Hematopoietic Stem Cells. Genes Dev..

[21] Li Y., Li Y., Zhang B., Zhao J., Qin J., Jiang S., Li Y., Chen Y., Li J., Chen K. (2025). Single-Cell Transcriptomics Reveals BMP4-BMPR2 Signaling Promotes Radiation Resistance in Hematopoietic Stem Cells Following Injury. Nat. Commun..

[22] Kim J.-H., Thimmulappa R.K., Kumar V., Cui W., Kumar S., Kombairaju P., Zhang H., Margolick J., Matsui W., Macvittie T. (2014). NRF2-Mediated Notch Pathway Activation Enhances Hematopoietic Reconstitution Following Myelosuppressive Radiation. J. Clin. Investig..

[23] Poulos M.G., Ramalingam P., Gutkin M.C., Kleppe M., Ginsberg M., Crowley M.J.P., Elemento O., Levine R.L., Rafii S., Kitajewski J. (2016). Endothelial-Specific Inhibition of NF-κB Enhances Functional Haematopoiesis. Nat. Commun..

[24] Hoppe P.S., Schwarzfischer M., Loeffler D., Kokkaliaris K.D., Hilsenbeck O., Moritz N., Endele M., Filipczyk A., Gambardella A., Ahmed N. (2016). Early Myeloid Lineage Choice Is Not Initiated by Random PU.1 to GATA1 Protein Ratios. Nature.

[25] Orkin S.H., Zon L.I. (2008). Hematopoiesis: An Evolving Paradigm for Stem Cell Biology. Cell.

[26] Gavriilidis G.I., Vasileiou V., Orfanou A., Ishaque N., Psomopoulos F. (2024). A Mini-Review on Perturbation Modelling across Single-Cell Omic Modalities. Comput. Struct. Biotechnol. J..

[27] Ji Y., Lotfollahi M., Wolf F.A., Theis F.J. (2021). Machine Learning for Perturbational Single-Cell Omics. Cell Syst..

[28] Kamimoto K., Stringa B., Hoffmann C.M., Jindal K., Solnica-Krezel L., Morris S.A. (2023). Dissecting Cell Identity via Network Inference and in Silico Gene Perturbation. Nature.

[29] Bravo González-Blas C., De Winter S., Hulselmans G., Hecker N., Matetovici I., Christiaens V., Poovathingal S., Wouters J., Aibar S., Aerts S. (2023). SCENIC+: Single-Cell Multiomic Inference of Enhancers and Gene Regulatory Networks. Nat. Methods.

[30] Burkhardt D.B., Stanley J.S., Tong A., Perdigoto A.L., Gigante S.A., Herold K.C., Wolf G., Giraldez A.J., van Dijk D., Krishnaswamy S. (2021). Quantifying the Effect of Experimental Perturbations at Single-Cell Resolution. Nat. Biotechnol..

[31] Aibar S., González-Blas C.B., Moerman T., Huynh-Thu V.A., Imrichova H., Hulselmans G., Rambow F., Marine J.-C., Geurts P., Aerts J. (2017). SCENIC: Single-Cell Regulatory Network Inference and Clustering. Nat. Methods.

[32] Van de Sande B., Flerin C., Davie K., De Waegeneer M., Hulselmans G., Aibar S., Seurinck R., Saelens W., Cannoodt R., Rouchon Q. (2020). A Scalable SCENIC Workflow for Single-Cell Gene Regulatory Network Analysis. Nat. Protoc..

[33] Moriguchi T., Yamamoto M. (2014). A Regulatory Network Governing *Gata1* and *Gata2* Gene Transcription Orchestrates Erythroid Lineage Differentiation. Int. J. Hematol..

[34] Randrianarison-Huetz V., Laurent B., Bardet V., Blobe G.C., Huetz F., Duménil D. (2010). Gfi-1B Controls Human Erythroid and Megakaryocytic Differentiation by Regulating TGF-Beta Signaling at the Bipotent Erythro-Megakaryocytic Progenitor Stage. Blood.

[35] Wang W., Xia X., Mao L., Wang S. (2019). The CCAAT/Enhancer-Binding Protein Family: Its Roles in MDSC Expansion and Function. Front. Immunol..

[36] Shyamsunder P., Shanmugasundaram M., Mayakonda A., Dakle P., Teoh W.W., Han L., Kanojia D., Lim M.C., Fullwood M., An O. (2019). Identification of a Novel Enhancer of CEBPE Essential for Granulocytic Differentiation. Blood.

[37] Graf T. (1992). Myb: A Transcriptional Activator Linking Proliferation and Differentiation in Hematopoietic Cells. Curr. Opin. Genet. Dev..

[38] Singh A.K., Swarnalatha M., Kumar V. (2011). c-ETS1 Facilitates G_1_/S-Phase Transition by Up-Regulating Cyclin E and *CDK2* Genes and Cooperates with Hepatitis B Virus X Protein for Their Deregulation. J. Biol. Chem..

[39] John S., Kalathil D., Pothuraju R., Nair S.A. (2025). Deciphering ETS2: An Indispensable Conduit to Cancer. Biochim. Biophys. Acta (BBA)-Rev. Cancer.

[40] Amanda S., Tan T.K., Iida S., Sanda T. (2022). Lineage- and Stage-Specific Oncogenicity of IRF4. Exp. Hematol..

[41] Brune Z., Rice M.R., Barnes B.J. (2020). Potential T Cell-Intrinsic Regulatory Roles for IRF5 via Cytokine Modulation in T Helper Subset Differentiation and Function. Front. Immunol..

[42] Das T., Wang E., Xu Y., Yang H., Liao X., Jain M.K. (2025). Krüppel-like Factor 4 Control of Immune Cell Function. Front. Immunol..

[43] Kee B.L. (2020). It’s a Phase That EBF1 Is Going Through. Immunity.

[44] del Bosque-Plata L., Hernández-Cortés E.P., Gragnoli C. (2022). The Broad Pathogenetic Role of *TCF7L2* in Human Diseases beyond Type 2 Diabetes. J. Cell. Physiol..

[45] Trompouki E., Bowman T.V., Lawton L.N., Fan Z.P., Wu D.-C., DiBiase A., Martin C.S., Cech J.N., Sessa A.K., Leblanc J.L. (2011). Lineage Regulators Direct BMP and Wnt Pathways to Cell-Specific Programs During Differentiation and Regeneration. Cell.

[46] Xing S., Gai K., Li X., Shao P., Zeng Z., Zhao X., Zhao X., Chen X., Paradee W.J., Meyerholz D.K. (2019). Tcf1 and Lef1 Are Required for the Immunosuppressive Function of Regulatory T Cells. J. Exp. Med..

[47] Zohren F., Souroullas G.P., Luo M., Gerdemann U., Imperato M.R., Wilson N.K., Gottgens B., Lukov G.L., Goodell M.A. (2012). Lyl1 Regulates Lymphoid Specification and Maintenance of Early T Lineage Progenitors. Nat. Immunol..

[48] Jang Y., Feng R., Palmer L.E., Mayuranathan T., Yao Y., Mayberry K., Zhou S., Xu J., Gossett J.M., Kang G. (2025). BCL11A-Deficient Human Erythropoiesis Is Impaired in Vitro and after Xenotransplantation into Mice. Blood Adv..

[49] Braun T.P., Okhovat M., Coblentz C., Carratt S.A., Foley A., Schonrock Z., Curtiss B.M., Nevonen K., Davis B., Garcia B. (2019). Myeloid Lineage Enhancers Drive Oncogene Synergy in CEBPA/CSF3R Mutant Acute Myeloid Leukemia. Nat. Commun..

[50] Kuznetsova T., Prange K.H.M., Glass C.K., De Winther M.P.J. (2020). Transcriptional and Epigenetic Regulation of Macrophages in Atherosclerosis. Nat. Rev. Cardiol..

[51] Li M., Jiang P., Cheng K., Zhang Z., Lan S., Li X., Zhao L., Wang Y., Wang X., Chen J. (2021). Regulation of MYB by Distal Enhancer Elements in Human Myeloid Leukemia. Cell Death Dis..

[52] Lien C., Fang C.-M., Huso D., Livak F., Lu R., Pitha P.M. (2010). Critical Role of IRF-5 in Regulation of B-Cell Differentiation. Proc. Natl. Acad. Sci. USA.

[53] Hagman J., Ramírez J., Lukin K. (2012). B Lymphocyte Lineage Specification, Commitment and Epigenetic Control of Transcription by Early B Cell Factor 1. Epigenetic Regulation of Lymphocyte Development.

[54] Zhang J., Lyu T., Cao Y., Feng H. (2021). Role of TCF-1 in Differentiation, Exhaustion, and Memory of CD8^+^ T Cells: A Review. FASEB J..

[55] Horiuchi S., Koike T., Takebuchi H., Hoshino K., Sasaki I., Fukuda-Ohta Y., Kaisho T., Kitamura D. (2023). SpiB Regulates the Expression of B-Cell-Related Genes and Increases the Longevity of Memory B Cells. Front. Immunol..

[56] Jacobs-Helber S.M., Abutin R.M., Tian C., Bondurant M., Wickrema A., Sawyer S.T. (2002). Role of JunB in Erythroid Differentiation. J. Biol. Chem..

[57] Sidhu K., Kumar V. (2015). C-ETS Transcription Factors Play an Essential Role in the Licensing of Human MCM4 Origin of Replication. Biochim. Biophys. Acta.

[58] Ma X., Xu L., Alberobello A.T., Gavrilova O., Bagattin A., Skarulis M., Liu J., Finkel T., Mueller E. (2015). Celastrol Protects against Obesity and Metabolic Dysfunction through Activation of a HSF1-PGC1α Transcriptional Axis. Cell Metab..

[59] Zhu J., Sun X., Zhan Y. (2025). Targeting HSF1-TLR9 Axis: Celastrol as a Potential Therapeutic for Liver Injury in Traumatic Hemorrhagic Shock. Discov. Med..

[60] Dong Q., Xiu Y., Wang Y., Hodgson C., Borcherding N., Jordan C., Buchanan J., Taylor E., Wagner B., Leidinger M. (2022). HSF1 Is a Driver of Leukemia Stem Cell Self-Renewal in Acute Myeloid Leukemia. Nat. Commun..

[61] Cabal-Hierro L., van Galen P., Prado M.A., Higby K.J., Togami K., Mowery C.T., Paulo J.A., Xie Y., Cejas P., Furusawa T. (2020). Chromatin Accessibility Promotes Hematopoietic and Leukemia Stem Cell Activity. Nat. Commun..

[62] Swift M.L., Beishline K., Flashner S., Azizkhan-Clifford J. (2021). DSB Repair Pathway Choice Is Regulated by Recruitment of 53BP1 through Cell Cycle-Dependent Regulation of Sp1. Cell Rep..

[63] Shields B.J., Jackson J.T., Metcalf D., Shi W., Huang Q., Garnham A.L., Glaser S.P., Beck D., Pimanda J.E., Bogue C.W. (2016). Acute Myeloid Leukemia Requires Hhex to Enable PRC2-Mediated Epigenetic Repression of *Cdkn2a*. Genes Dev..

[64] Fang Y., Cheng L., Huang M., Cao Y., Zou Q., Cai J., Zhang Y., Xia Y., Huang H., Chen X. (2025). Heat Shock Factor 1 Promotes Proliferation and Chemoresistance in Diffuse Large B-Cell Lymphoma by Enhancing the Cell Cycle and DNA Repair. Cell Death Dis..

[65] Li J., Wang Q.-E., Zhu Q., El-Mahdy M.A., Wani G., Praetorius-Ibba M., Wani A.A. (2006). DNA Damage Binding Protein Component DDB1 Participates in Nucleotide Excision Repair through DDB2 DNA-Binding and Cullin 4A Ubiquitin Ligase Activity. Cancer Res..

[66] Wenger J., McGrath K.E., Koniski A., Cacciatori J., Bushnell T.P., Dertinger S.D., Chen Y., Palis J. (2007). Response of the Erythroid Lineage to Irradiation. Blood.

[67] Wang J., Sun Q., Morita Y., Jiang H., Groß A., Lechel A., Hildner K., Guachalla L.M., Gompf A., Hartmann D. (2012). A Differentiation Checkpoint Limits Hematopoietic Stem Cell Self-Renewal in Response to DNA Damage. Cell.

[68] Li W., Wang X., Dong Y., Huo Q., Yue T., Wu X., Lu L., Zhang J., Zhao Y., Dong H. (2023). Nicotinamide Riboside Intervention Alleviates Hematopoietic System Injury of Ionizing Radiation-induced Premature Aging Mice. Aging Cell.

[69] Liao W., Liu C., Yang K., Chen J., Wu Y., Zhang S., Yu K., Wang L., Ran L., Chen M. (2023). Aged Hematopoietic Stem Cells Entrap Regulatory T Cells to Create a Prosurvival Microenvironment. Cell. Mol. Immunol..

[70] Kurashina R., Ohyashiki J.H., Kobayashi C., Hamamura R., Zhang Y., Hirano T., Ohyashiki K. (2009). Anti-Proliferative Activity of Heat Shock Protein (Hsp) 90 Inhibitors via Beta-Catenin/TCF7L2 Pathway in Adult T Cell Leukemia Cells. Cancer Lett..

[71] Jain N., Hartert K., Tadros S., Fiskus W., Havranek O., Ma M.C.J., Bouska A., Heavican T., Kumar D., Deng Q. (2019). Targetable Genetic Alterations of *TCF4* (*E2-2*) Drive Immunoglobulin Expression in Diffuse Large B Cell Lymphoma. Sci. Transl. Med..

[72] Macias A., Cortes M., An N., Li N., Fulton T., Simpson A., Miller S., Sun S., Fu D., Garcia J. (2023). From in Silico to in Vitro to In Vivo: Increasing Megakaryocytes to Treat Thrombocytopenia. Blood.

[73] Sharma S., Mishra R., Walker B.L., Deshmukh S., Zampino M., Patel J., Anamalai M., Simpson D., Singh I.S., Kaushal S. (2015). Celastrol, an Oral Heat Shock Activator, Ameliorates Multiple Animal Disease Models of Cell Death. Cell Stress Chaperones.

[74] Kruta M., Sunshine M.J., Chua B.A., Fu Y., Chawla A., Dillingham C.H., Hidalgo San Jose L., De Jong B., Zhou F.J., Signer R.A.J. (2021). Hsf1 Promotes Hematopoietic Stem Cell Fitness and Proteostasis in Response to Ex Vivo Culture Stress and Aging. Cell Stem Cell.

[75] Kim Y.J., Lam K., Zhou F., Ong C.M., Magee J.A., Signer R. (2022). A Stress Response Pathway That Enhances Hematopoietic Stem Cell Longevity Promotes Acute Myeloid Leukemia Growth and Progression. Blood.

[76] Li Q., Martinez J.D. (2011). Loss of HSF1 Results in Defective Radiation-Induced G2 Arrest and DNA Repair. Radiat. Res..

[77] Chin Y., Gumilar K.E., Li X.-G., Tjokroprawiro B.A., Lu C.-H., Lu J., Zhou M., Sobol R.W., Tan M. (2023). Targeting HSF1 for Cancer Treatment: Mechanisms and Inhibitor Development. Theranostics.

[78] Luan H., Yang J., Wang Y., Shen X., Zhang X., Qiao Z., Xing S., Yu Z. (2023). rhTPO Ameliorates Radiation-Induced Long-Term Hematopoietic Stem Cell Injury in Mice. Molecules.

[79] Acharya S.S., Fendler W., Watson J., Hamilton A., Pan Y., Gaudiano E., Moskwa P., Bhanja P., Saha S., Guha C. (2015). Serum microRNAs Are Early Indicators of Survival after Radiation-Induced Hematopoietic Injury. Sci. Transl. Med..

[80] Xu L.-N., Zhao N., Chen J.-Y., Ye P.-P., Nan X.-W., Zhou H.-H., Jiang Q.-W., Yang Y., Huang J.-R., Yuan M.-L. (2019). Celastrol Inhibits the Growth of Ovarian Cancer Cells in Vitro and in Vivo. Front. Oncol..

[81] Zhu B., Wei Y. (2019). Antitumor Activity of Celastrol by Inhibition of Proliferation, Invasion, and Migration in Cholangiocarcinoma via PTEN/PI3K/Akt Pathway. Cancer Med..

[82] Zhang X., Yang J., Chen M., Li L., Huan F., Li A., Liu Y., Xia Y., Duan J., Ma S. (2016). Metabolomics Profiles Delineate Uridine Deficiency Contributes to Mitochondria-Mediated Apoptosis Induced by Celastrol in Human Acute Promyelocytic Leukemia Cells. Oncotarget.

[83] Hao Y., Hao S., Andersen-Nissen E., Mauck W.M., Zheng S., Butler A., Lee M.J., Wilk A.J., Darby C., Zager M. (2021). Integrated Analysis of Multimodal Single-Cell Data. Cell.

[84] Wolock S.L., Lopez R., Klein A.M. (2019). Scrublet: Computational Identification of Cell Doublets in Single-Cell Transcriptomic Data. Cell Syst..

[85] Hänzelmann S., Castelo R., Guinney J. (2013). GSVA: Gene Set Variation Analysis for Microarray and RNA-Seq Data. BMC Bioinform..

[86] Liberzon A., Birger C., Thorvaldsdóttir H., Ghandi M., Mesirov J.P., Tamayo P. (2015). The Molecular Signatures Database (MSigDB) Hallmark Gene Set Collection. Cell Syst..

[87] Shannon P., Markiel A., Ozier O., Baliga N.S., Wang J.T., Ramage D., Amin N., Schwikowski B., Ideker T. (2003). Cytoscape: A Software Environment for Integrated Models of Biomolecular Interaction Networks. Genome Res..

[88] Langmead B., Salzberg S.L. (2012). Fast Gapped-Read Alignment with Bowtie 2. Nat. Methods.

[89] Zhang Y., Liu T., Meyer C.A., Eeckhoute J., Johnson D.S., Bernstein B.E., Nusbaum C., Myers R.M., Brown M., Li W. (2008). Model-Based Analysis of ChIP-Seq (MACS). Genome Biol..

[90] Heinz S., Benner C., Spann N., Bertolino E., Lin Y.C., Laslo P., Cheng J.X., Murre C., Singh H., Glass C.K. (2010). Simple Combinations of Lineage-Determining Transcription Factors Prime Cis-Regulatory Elements Required for Macrophage and B Cell Identities. Mol. Cell.

[91] Ramírez F., Dündar F., Diehl S., Grüning B.A., Manke T. (2014). deepTools: A Flexible Platform for Exploring Deep-Sequencing Data. Nucleic Acids Res..

[92] Thorvaldsdóttir H., Robinson J.T., Mesirov J.P. (2013). Integrative Genomics Viewer (IGV): High-Performance Genomics Data Visualization and Exploration. Brief. Bioinform..

[93] Wu T., Hu E., Xu S., Chen M., Guo P., Dai Z., Feng T., Zhou L., Tang W., Zhan L. (2021). clusterProfiler 4.0: A Universal Enrichment Tool for Interpreting Omics Data. Innovation.

[94] Yang P., Chen X., Wen H., Yu M., Yu H., Wang L., Gong L., Zhao L. (2025). In Vitro Differentiation of Common Lymphoid Progenitor Cells into B Cell Using Stromal Cell Free Culture System. BMC Immunol..

[95] Tang D., Tao S., Chen Z., Koliesnik I.O., Calmes P.G., Hoerr V., Han B., Gebert N., Zörnig M., Löffler B. (2016). Dietary Restriction Improves Repopulation but Impairs Lymphoid Differentiation Capacity of Hematopoietic Stem Cells in Early Aging. J. Exp. Med..

[96] Chen T., Chen X., Zhang S., Zhu J., Tang B., Wang A., Dong L., Zhang Z., Yu C., Sun Y. (2021). The Genome Sequence Archive Family: Toward Explosive Data Growth and Diverse Data Types. Genom. Proteom. Bioinform..

[97] Zou Z., Ohta T., Oki S. (2024). ChIP-Atlas 3.0: A Data-Mining Suite to Explore Chromosome Architecture Together with Large-Scale Regulome Data. Nucleic Acids Res..

[98] Zheng R., Wan C., Mei S., Qin Q., Wu Q., Sun H., Chen C.-H., Brown M., Zhang X., Meyer C.A. (2019). Cistrome Data Browser: Expanded Datasets and New Tools for Gene Regulatory Analysis. Nucleic Acids Res..

